# Nanoparticles presenting clusters of CD4 expose a universal vulnerability of HIV-1 by mimicking target cells

**DOI:** 10.1073/pnas.2010320117

**Published:** 2020-07-20

**Authors:** Magnus A. G. Hoffmann, Yotam Bar-On, Zhi Yang, Harry B. Gristick, Priyanthi N. P. Gnanapragasam, Jost Vielmetter, Michel C. Nussenzweig, Pamela J. Bjorkman

**Affiliations:** ^a^Division of Biology and Biological Engineering, California Institute of Technology, Pasadena, CA 91125;; ^b^Laboratory of Molecular Immunology, Rockefeller University, New York, NY 10065

**Keywords:** HIV-1, decoy therapeutics, virus-like particles, functional cure

## Abstract

CD4-based decoy approaches against HIV-1 are attractive options for long-term viral control, but initial designs, including soluble CD4 (sCD4) and CD4-Ig, were ineffective. To evaluate a therapeutic that more accurately mimics HIV-1 target cells compared with monomeric sCD4 and dimeric CD4-Ig, we generated virus-like nanoparticles that present clusters of membrane-associated CD4 (CD4-VLPs) to permit high-avidity binding of trimeric HIV-1 envelope spikes. In neutralization assays, CD4-VLPs were >12,000-fold more potent than sCD4 and CD4-Ig and >100-fold more potent than the broadly neutralizing antibody (bNAb) 3BNC117, with >12,000-fold improvements against strains poorly neutralized by 3BNC117. CD4-VLPs also neutralized patient-derived viral isolates that were resistant to 3BNC117 and other bNAbs. Intraperitoneal injections of CD4-CCR5-VLP produced only subneutralizing plasma concentrations in HIV-1–infected humanized mice but elicited CD4-binding site mutations that reduced viral fitness. All mutant viruses showed reduced sensitivity to sCD4 and CD4-Ig but remained sensitive to neutralization by CD4-VLPs in vitro. In vitro evolution studies demonstrated that CD4-VLPs effectively controlled HIV-1 replication at neutralizing concentrations, and viral escape was not observed. Moreover, CD4-VLPs potently neutralized viral swarms that were completely resistant to CD4-Ig, suggesting that escape pathways that confer resistance against conventional CD4-based inhibitors are ineffective against CD4-VLPs. These findings suggest that therapeutics that mimic HIV-1 target cells could prevent viral escape by exposing a universal vulnerability of HIV-1: the requirement to bind CD4 on a target cell. We propose that therapeutic and delivery strategies that ensure durable bioavailability need to be developed to translate this concept into a clinically feasible functional cure therapy.

## Significance

The ability of HIV-1 to rapidly develop resistance against various therapeutics remains a roadblock to finding a cure. Here we propose that nanoparticle-based therapeutics that mimic HIV-1 target cells by presenting clusters of CD4, the HIV-1 receptor, could prevent effective viral escape. We show that CD4 multimerization on the nanoparticle dramatically enhanced the neutralization potency and breadth compared with conventional CD4-based reagents that present only one or two copies of CD4. The CD4 nanoparticles neutralized a range of diverse HIV-1 strains, including patient isolates resistant to multiple broadly neutralizing antibodies. This work describes a new therapeutic direction that exploits the need of HIV-1 to bind CD4 on target cells, thus warranting further investigation for the potential development of a cure.

Antiretroviral therapy (ART) prolongs the life expectancy of HIV-1–infected individuals but is associated with side effects, and multiple drugs need to be given in combination to prevent the development of viral resistance (1). In addition, treatment must continue for the lifetime of the individual due to the existence of a long-lived latent proviral reservoir. While a “sterilizing” cure remains difficult to achieve due to difficulties associated with identifying and clearing latently infected cells (2, 3), recent research has focused on designing a “functional” cure, i.e., a therapeutic strategy that enables long-term suppression of HIV-1 replication and remission of symptoms in the absence of ART (4).

The development of viral resistance is a major obstacle to achieving a functional cure, since low levels of latent replication-competent viruses persist in the body. Decoy approaches that closely mimic HIV-1 target cells are attractive options for long-term viral control, as viral resistance through mutation cannot develop without concomitant loss of target cell infectivity (5, 6). HIV-1 primarily infects CD4^+^ T cells; the gp120 subunit of the viral envelope glycoprotein (Env) initially binds CD4, triggering a conformational change that allows it to interact with a host cell coreceptor protein, the chemokine receptor CCR5 or CXCR4, leading to fusion between the viral and host cell membranes (7).

**Fig. 1.**
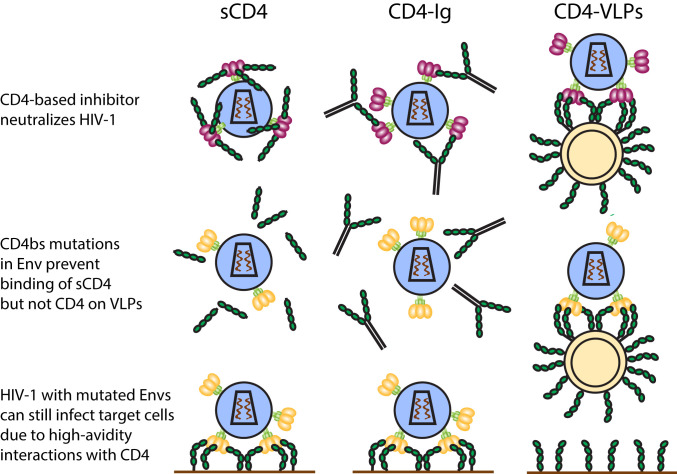
CD4-VLPs can overcome HIV-1 Env mutations that prevent neutralization by soluble CD4-based inhibitors. Schematic comparison of responses of soluble CD4-based inhibitors and CD4-VLPs to Env mutations. Monovalent sCD4 and bivalent CD4-Ig neutralize sCD4-sensitive strains (purple Env trimers; row 1), but mutations in the CD4bs that lower the affinity of Env for sCD4 (yellow Env trimers; row 2) render other strains resistant to soluble CD4-based inhibitors while maintaining the ability to infect CD4^+^ target cells via avidity effects through binding to multiple CD4 receptors tethered to the host cell membrane (row 3). CD4-VLPs can neutralize viral strains that are resistant to soluble CD4-based inhibitors through high-avidity binding to multiple CD4 receptors tethered to the VLP membrane.

Initial attempts to design decoys against HIV-1 used a soluble form of CD4 (sCD4) to block the receptor-binding sites on Env, but this strategy proved ineffective in patients (8, 9). Subsequent studies revealed that many primary HIV-1 isolates were relatively insensitive to sCD4 neutralization without apparent loss of viral fitness (10). In addition, HIV-1 can develop resistance to CD4-based inhibitors by acquiring mutations in the CD4-binding site (CD4bs) on gp120 that lower its affinity for CD4 (11–13) (Fig. 1). A potential explanation for the shortcomings of sCD4 therapy is that monomeric sCD4 fails to accurately mimic an HIV-1 target cell in which clusters of CD4 molecules on the membrane could enable Env, a trimeric protein with three CD4bs, to form multiple interactions that tether it to the cell surface (14). Thus, HIV-1 variants could escape from sCD4-mediated inhibition through avidity effects that compensate for a lower intrinsic sCD4-binding affinity by using multivalent interactions, thereby retaining the ability to efficiently infect target cells. Dimeric CD4-Ig fusion proteins (CD4-Ig) (15) do not overcome this problem, since bivalent binding with both CD4 arms is prevented by the low density of Env spikes on the viral surface and/or the architecture of single Env trimers (16–18) (Fig. 1).

To test this hypothesis, we generated HIV-1 Gag-based virus-like nanoparticles that present clusters of CD4 in its natural membrane-associated conformation (CD4-VLPs) (Fig. 1). We demonstrate that CD4-VLPs neutralize HIV-1 with enhanced potency and breadth compared with sCD4, CD4-Ig, and 3BNC117, a broadly neutralizing antibody (bNAb) that targets the CD4bs. We also show that viral escape pathways that confer resistance to sCD4 and CD4-Ig are ineffective against CD4-VLPs, suggesting that therapeutics that mimic HIV-1 target cells could prevent viral escape by exposing a universal vulnerability, the requirement to bind clusters of CD4 on a target cell.

## Results

### CD4-VLPs Potently Neutralize HIV-1.

**Fig. 2.**
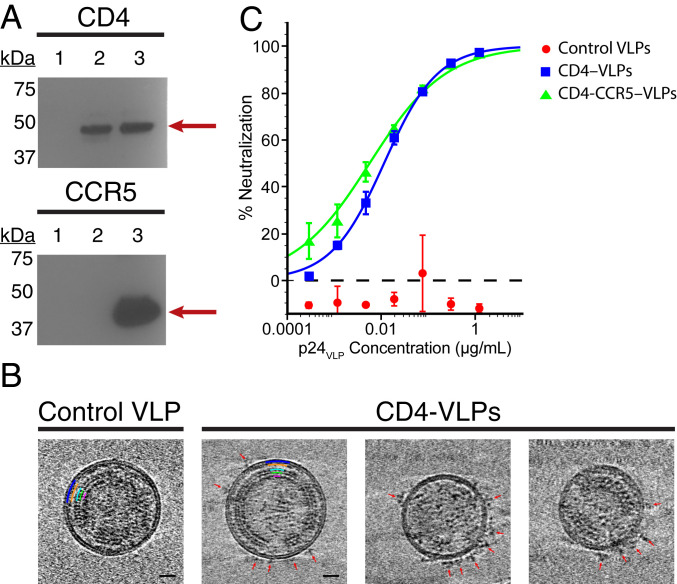
VLPs incorporate CD4 and CCR5 and neutralize HIV-1. (*A*) Western blot analysis to detect CD4 (*Left*) and CCR5 (*Right*) in supernatants containing HIV-1 Gag-derived VLPs from Expi293 cells transfected with Gag alone (control VLPs; lane 1), Gag and CD4 (CD4-VLPs; lane 2), Gag, CD4, and CCR5 (CD4-CCR5-VLPs; lane 3). (*B*) Tomographic slices (10.9 nm) from cryo-ET analysis of control VLPs (*Left*) or CD4-VLPs (*Right* three panels) incubated with a soluble native-like Env trimer (BG505 SOSIP.664). (Scale bar: 20 nm.) Red arrows indicate densities for bound Env trimers. Approximate positions of immature Gag shell layers are highlighted in the *Left* two panels. Note the hexagonal lattice in the capsid layer of Gag. See also Movie S1. (*C*) In vitro neutralization assay against HIV-1_YU2_ pseudovirus comparing control VLPs, CD4-VLPs, and CD4-CCR5-VLPs. The neutralization activity of VLPs was determined in terms of concentrations of the Gag p24 capsid protein. Data points are presented as the mean and SD of duplicate measurements.

CD4-VLPs were generated by transiently coexpressing HIV-1 Gag and CD4 in Expi293 cells. When expressed in human cells, the Gag polyprotein self-assembles into immature core particles that form ∼120-nm-diameter VLPs by budding through the plasma membrane (19). CD4-CCR5-VLPs were also generated to investigate whether adding a coreceptor would enhance the potency and breadth of HIV-1 neutralization. VLPs were collected from transfected cell supernatants and concentrated by centrifugal filtration or sucrose cushion ultracentrifugation (*Methods*). Western blot analysis confirmed that CD4 and CCR5 were present on CD4-VLPs and CD4-CCR5-VLPs, respectively, but not on control VLPs (Fig. 2*A*). To determine VLP concentrations, we converted p24 measurements from ELISAs against the Gag p24 capsid protein into VLP concentrations by assuming that each VLP contains 2,000 copies of Gag (20, 21) (*Methods*). Typical VLP concentrations in transfected supernatants were ∼10^10^ VLPs/mL, which could be concentrated to ∼10^11^ VLPs/mL. To estimate the number of CD4 molecules per VLP, we combined supernatants from five independent CD4-VLP productions and purified CD4-VLPs using sucrose cushion ultracentrifugation and size exclusion chromatography (*SI Appendix*, Fig. S1 *A* and *B*). Quantitative Western blot analysis showed that purified CD4-VLPs contained the Gag-EGFP fusion protein and CD4 at a molar ratio of ∼14:1 (*SI Appendix*, Fig. S2 *A*–*C*), suggesting that CD4-VLPs incorporated an average of 140 ± 48 molecules of CD4. Cryo-electron tomography (cryo-ET) imaging of purified CD4-VLPs revealed spherical particles of ∼120 nm diameter with discernible internal layers of immature Gag (Fig. 2*B* and Movie S1). Although membrane-bound CD4 molecules are too small to be visualized by cryo-ET, their presence on a number of CD4-VLPs, but not on control VLPs, was confirmed by densities for bound soluble Env trimers (Fig. 2*B* and Movie S1). However, since there is no specific mechanism for packaging CD4 and CCR5 into HIV-1 Gag-based VLPs, CD4-VLPs containing little or no CD4 were also observed.

The ability of CD4-VLPs to inhibit HIV-1 infection of target cells was evaluated using pseudovirus-based TZM-bl neutralization assays (22). CD4-VLPs and CD4-CCR5-VLPs neutralized the HIV-1 strain YU2 at half maximal inhibitory concentrations (IC_50_s) of 0.012 μg p24/mL and 0.006 μg p24/mL, equivalent to 1.5 × 10^8^ and 0.8 × 10^8^ VLPs/mL, respectively (Fig. 2*C*). Control VLPs showed no neutralization at concentrations up to 1.5 × 10^10^ VLPs/mL. Neutralization activity was independent of the VLP purification method and similar for different batches of CD4-VLPs (*SI Appendix*, Fig. S3). A concentration of 2.1 × 10^10^ CD4 molecules/mL (0.0017 μg/mL) (calculated assuming that each CD4-VLP displays 140 copies of CD4) was required to achieve 50% neutralization. The comparable neutralization profiles of CD4-VLPs and CD4-CCR5-VLPs suggested that the presence of CD4 on VLPs was sufficient for potent HIV-1 neutralization in the absence of CCR5. Extracellular vesicles naturally secreted by eukaryotic cells (23) did not contribute to the neutralization activity of CD4-VLPs, as supernatants from cells transfected with CD4 in the absence of Gag had no effect (*SI Appendix*, Fig. S4 *A* and *B*).

### CD4-VLPs Neutralize HIV-1 with Enhanced Potency and Breadth Compared with sCD4, CD4-Ig, and a CD4bs bNAb.

To investigate whether CD4 multimerization enhances the potency and breadth of CD4-based inhibitors, the neutralization activity of CD4-VLPs was compared with that of monovalent sCD4, bivalent CD4-Ig, and 3BNC117 IgG, a CD4bs bNAb that has been evaluated in human clinical trials (24, 25). Potency and breadth were compared by quantifying the number of CD4 molecules (CD4-VLPs, sCD4, CD4-Ig) or antigen-binding fragments (Fabs; 3BNC117) required to neutralize a panel of 12 HIV-1 Env reference strains representing the global HIV-1 epidemic (26).

**Fig. 3.**
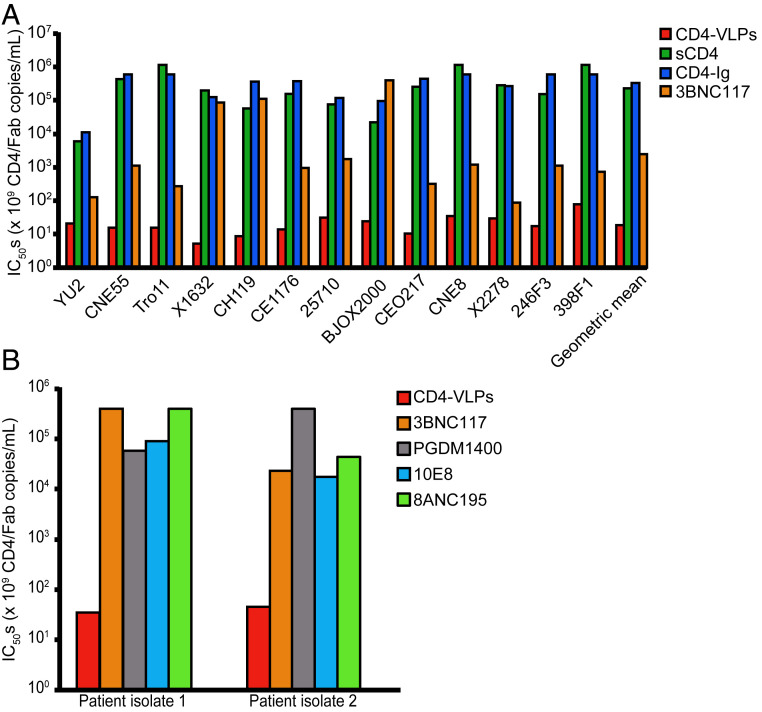
CD4-VLPs neutralize HIV-1 with enhanced potency and breadth compared with sCD4, CD4-Ig, and 3BNC117. (*A*) In vitro neutralization of HIV-1_YU2_ and a panel of 12 HIV-1 Env reference strains (26). IC_50_ values against each strain and the geometric mean IC_50_ are presented as CD4 copies/mL for CD4-VLPs, sCD4, and CD4-Ig and as Fab copies/mL for 3BNC117 (*SI Appendix*, Tables S1 and S2). IC_50_ values of 50 μg/mL were used for strains that were not neutralized at 50 μg/mL by sCD4, CD4-Ig, and 3BNC117 for the geometric mean IC_50_ calculations. (*B*) In vitro neutralization of two HIV-1 patient isolates that exhibited resistance against multiple bNAbs (27). IC_50_ values are presented in CD4 copies/mL for CD4-VLPs and in Fab copies/mL for 3BNC117 (CD4bs bNAb), PGDM1400 (V1V2 bNAb), 10E8 (membrane-proximal external region bNAb), and 8ANC195 (gp120–gp41 interface bNAb) (*SI Appendix*, Table S3).

CD4-VLPs neutralized all 12 strains with a geometric mean IC_50_ of 1.3 × 10^8^ VLPs/mL (*SI Appendix*, Table S1). CD4-CCR5-VLPs did not show an overall enhanced potency compared with CD4-VLPs (geometric mean IC_50_ = 1.2 × 10^8^ VLPs/mL). Assuming ∼140 copies of CD4 per VLP (*SI Appendix*, Fig. S2 *A*–*C*), neutralization was achieved at a geometric mean IC_50_ of 1.9 × 10^10^ CD4 molecules/mL (Fig. 3*A* and *SI Appendix*, Table S1). sCD4 and CD4-Ig neutralized only nine (sCD4) or seven (CD4-Ig) of 12 strains with geometric mean IC_50_ values of 10.0 and 27.7 μg/mL, respectively, equivalent to 2.3 × 10^14^ and 3.3 × 10^14^ CD4 molecules/mL (Fig. 3*A* and *SI Appendix*, Table S2). These results demonstrate that CD4-mediated neutralization of HIV-1 is >12,000-fold more potent for multivalent CD4, likely due to high-avidity interactions between clustered CD4 receptors and HIV-1 Env.

CD4-VLPs were also more potent and broad than 3BNC117, which neutralized 11 of 12 strains with a geometric mean IC_50_ of 0.31 μg/mL (2.5 × 10^12^ Fabs per mL) (Fig. 3*A* and *SI Appendix*, Table S2). Thus, the geometric mean IC_50_ of CD4 molecules on CD4-VLPs was >100-fold lower than the required concentration of 3BNC117 Fabs and >12,000-fold lower for the X1632, CH119, and BJOX2000 strains, which are relatively insensitive to 3BNC117. Interestingly, there was only a 15-fold difference between the highest and lowest IC_50_ values against the 12 strains for CD4-VLPs, compared with a 4,500-fold difference for 3BNC117, highlighting the excellent neutralization breadth of CD4-VLPs.

The neutralization activity of CD4-VLPs was also evaluated against primary isolates obtained from two previously described HIV-1–infected patients (27). These isolates were poorly neutralized by bNAbs targeting various epitopes on Env, including the CD4bs, V1V2, the membrane-proximal external region, and the gp120–gp41 interface (IC_50_ >1 μg/mL) (Fig. 3*B* and *SI Appendix*, Table S3). However, both isolates were potently neutralized by CD4-VLPs at concentrations similar to IC_50_ values determined against the HIV-1 strains in the 12-strain panel. Comparing the numbers of CD4 and Fab molecules required for 50% neutralization revealed that CD4-VLPs were 500- to 11,000-fold more potent than the CD4bs bNAb 3BNC117. Similar differences in potency were also observed when comparing CD4-VLPs with bNAbs targeting epitopes other than the CD4bs. These results demonstrate that primary HIV-1 isolates that are resistant to multiple bNAbs can be potently neutralized by a therapeutic that mimics HIV-1 target cells.

### CD4-CCR5-VLP Treatment in HIV-1–Infected Hu-Mice Elicits CD4bs Mutations in HIV-1 Env Despite Poor Bioavailability.

We investigated the ability of CD4-CCR5-VLPs to suppress HIV-1 replication and prevent viral escape in vivo in HIV-1_YU2_–infected humanized mice (hu-mice). CD4-CCR5-VLPs were selected for in vivo experiments to prevent the potential emergence of CD4-independent HIV-1 escape variants (28–30). To determine an optimal administration regimen, initial half-life studies were performed in uninfected hu-mice. Here, 7.6 × 10^9^ CD4-CCR5-VLPs (610 ng of p24) were intraperitoneally (IP) injected into five hu-mice. Blood samples were taken from one animal per time point after 20 min, 1 h, 2 h, 4 h, and 6 h, and plasma CD4-CCR5-VLP concentrations were measured by p24 ELISA. CD4-CCR5-VLP concentrations were either slightly above or slightly below the detection limit of 1.5 ng p24/mL (1.9 × 10^7^ VLPs/mL) at all time points. A peak concentration of 8.5 × 10^7^ VLPs/mL was measured at 1 h postinjection. Incomplete diffusion across the peritoneal membrane into the hepatic portal vein and rapid hepatic clearance (31) likely contributed to the poor bioavailability of CD4-CCR5-VLPs administered IP. These studies showed that IP administrations of CD4-CCR5-VLPs reached only subneutralizing plasma concentrations, as peak levels were lower than in vitro neutralization IC_50_ values against HIV-1_YU2_ (*SI Appendix*, Table S1).

**Fig. 4.**
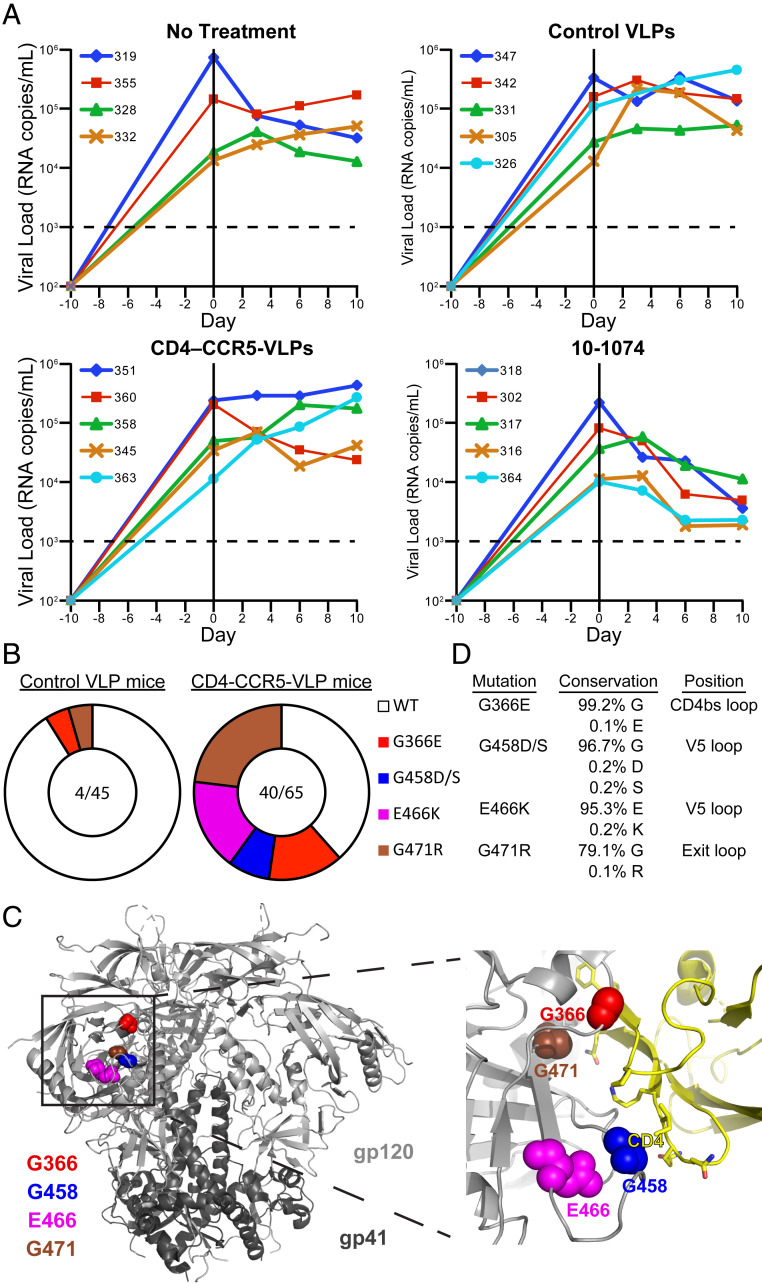
CD4-CCR5-VLP treatment in HIV-1_YU2_–infected hu-mice elicits CD4bs mutations in gp120. (*A*) Viral loads (RNA copies/mL) measured over time (days) in HIV-1_YU2_–infected hu-mice. Each line represents measurements for a single hu-mouse (identified by different numbers). The dotted line indicates the detection limit. Hu-mice were infected 10 d before initiation of treatment: no treatment, twice-daily IP injections of control VLPs (6 × 10^9^ control VLPs per injection), twice-daily IP injections of CD4-CCR5-VLPs (6 × 10^9^ VLPs per injection), or twice-weekly IP injections of the 10–1074 bNAb (1 mg per injection) for 10 d. (*B*) Pie charts showing the frequency of recurrent mutations in the gp120 subunits of the HIV-1_YU2_ *env* genes of plasma viruses obtained from control VLP-treated and CD4-CCR5-VLP–treated hu-mice. The slices are proportional to the number of sequences containing the indicated substitutions. White slices represent the number of sequences that lacked any recurrent mutations. The numbers in the center indicate the total number of sequences containing recurrent mutations over the total number of sequences analyzed. (*C*, *Left*) Cartoon diagram of Env trimer structure (Protein Data Bank ID code 5T3Z), with locations of residues that were mutated highlighted as colored surfaces. (*C*, *Right*) Close-up view of the gp120–CD4 binding interface highlighting the proximity of the mutated residues to the CD4bs. (*D*) Levels of conservation of each mutated residue and its respective substitution (https://www.hiv.lanl.gov/).

To achieve maximal plasma concentrations for experiments in HIV-1_YU2_–infected mice, 6 × 10^9^ control or CD4-CCR5-VLPs were injected IP twice daily for 10 d. This regimen failed to maintain detectable plasma VLP concentrations, as control VLPs and CD4-CCR5-VLPs were undetectable at 6 h postinjection on day 6 of treatment. The efficacy of CD4-CCR5-VLP treatment was compared with that of twice-weekly IP injections of 1 mg of 10–1074, a V3-glycan patch bNAb currently being evaluated in human clinical trials (32). While 10–1074 treatment achieved robust reductions in viral loads in all treated animals, control and CD4-CCR5-VLPs had no effect on viral loads (Fig. 4*A*).

To investigate whether intermittent subneutralizing CD4-CCR5-VLP plasma concentrations exert selective pressure on HIV-1 in vivo, the circulating plasma viruses of two HIV-1–infected control VLP-treated animals and three CD4-CCR5-VLP–treated animals were analyzed by single-genome sequencing on day 10 after treatment cessation. Four recurring mutations—G366E, G458D/S, E466K, and G471R—were observed in the gp120 subunits of the HIV-1_YU2_ Envs obtained from CD4-CCR5-VLP–treated hu-mice but were rare or absent from sequences derived from control VLP-treated animals (Fig. 4*B*). All individual mutations occurred in at least two animals and were mutually exclusive, except for one gp120 sequence that contained both G458D and G471R mutations. Together, these variants accounted for 61.5% of the *env* genes sequenced from CD4-CCR5-VLP–treated hu-mice, indicating that these mutations provided a selective advantage in the presence of intermittent subneutralizing CD4-CCR5-VLP plasma concentrations. Interestingly, in one CD4-CCR5-VLP–treated mouse, these mutations were found in 75% of the viruses (*SI Appendix*, Fig. S5).

The substitutions mapped to residues in close proximity to the CD4bs in the gp120 subunit of Env (Fig. 4*C*), suggesting that the mutations reduced the ability of Env to bind CD4, thereby potentially allowing escape from CD4-based reagents. Indeed, the G366E (33), G458D (34), and G471R (13) mutations have been reported to confer partial resistance against CD4-based inhibitors. No recurrent mutations were found near the coreceptor-binding site, indicating that selective pressure was exerted primarily by CD4 on the CD4-CCR5-VLPs. All mutated residues are highly conserved among HIV-1 Env sequences, whereas the substitutions in the variants are rare (Fig. 4*D*), suggesting that these mutations could compromise viral fitness.

### HIV-1_YU2_ Variants Are Less Infectious and Not Resistant to CD4-VLPs.

Surface plasmon resonance (SPR) studies were performed to determine whether the recurring mutations in *env* sequences from HIV-1–infected and CD4-CCR5-VLP–treated animals affected the CD4-binding affinity of HIV-1_YU2_ gp120. Potential avidity effects were avoided by injecting monomeric gp120 proteins (YU2_wt_ gp120 and each YU2 gp120 variant) over immobilized CD4-Ig (*SI Appendix*, Fig. S6*A*). The YU2_G366E_ gp120 mutant was excluded from the SPR analysis, as gel electrophoresis showed that this protein was unstable and migrated as multiple species (*SI Appendix*, Fig. S6*B*).

**Fig. 5.**
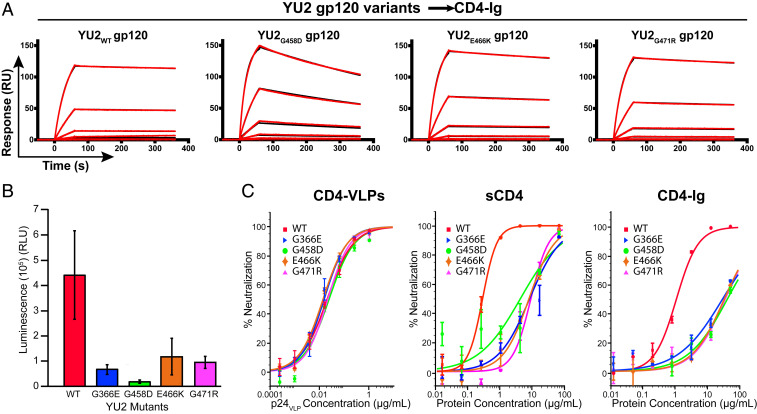
HIV-1_YU2_ variants have lower affinity for CD4, are less infectious, and are not resistant to CD4-VLPs. (*A*) SPR-binding assays of CD4-Ig with YU2_wt_ and YU2 variant gp120 proteins. Representative sensograms (red) and fits (black) for binding of YU2_wt_, YU2_G458D_, YU2_E466K_, and YU2_G471R_ gp120 proteins to CD4-Ig captured on a protein A biosensor chip (*SI Appendix*, Fig. S6*A*). The YU2_G366E_ gp120 mutant was excluded from the SPR analysis, as this protein was unstable (*SI Appendix*, Fig. S6*B*). YU2 gp120 proteins were flowed over the chip as a fourfold dilution series with a maximum concentration of 250 nM. K_D_, *k*_a_, and *k*_d_ values are presented in *SI Appendix*, Table S4. (*B*) Infectivity assay comparing the ability of WT and variant HIV-1_YU2_ pseudoviruses to enter and infect target cells. YU2_wt_ or mutant viruses (40 pg p24) were added to TZM-bl cells, and luminescence was measured after 48 h. Columns and error bars represent the mean and SDs for measurements from three separate experiments using eight replicates per experiment. (*C*) Overlay of neutralization curves for CD4-VLPs, sCD4, and CD4-Ig against YU2_wt_ and the indicated YU2 mutant pseudoviruses. Inhibitor concentrations are shown as p24 concentrations for CD4-VLPs (Fig. 2*C* and *Methods*) and protein concentrations for sCD4 and CD4-Ig. Data points are presented as the mean and SD of duplicate measurements. IC_50_ values for CD4-VLPs, CD4-CCR5-VLPs, sCD4, and CD4-Ig against YU2_wt_ and YU2 mutant pseudoviruses are presented in *SI Appendix*, Table S5.

Changes in CD4 binding were most evident for YU2_G458D_ gp120, which dissociated 11-fold faster than YU2_wt_ gp120 (Fig. 5*A* and *SI Appendix*, Table S4). Weaker binding of this mutant was expected, as the Gly in YU2_wt_ gp120 directly interacts with CD4, whereas the substituted Asp introduced a negative charge and potential steric clashes at this position (Fig. 4*C*). Changes in the CD4-binding affinity for the YU2_E466K_ and YU2_G471R_ gp120 mutants were less pronounced, since residues at both positions do not directly interact with CD4 (Fig. 4*C*). However, both substitutions introduced positive charges that could destabilize the CD4bs. Although SPR measurements were not possible for YU2_G366E_ gp120, the substitution introduces a larger side chain and a negative charge into a residue that directly contacts CD4, and thus it seems likely that YU2_G366E_ gp120 binds CD4 more weakly than YU2_wt_ gp120.

To ascertain whether the changes in CD4-binding affinity directly impacted the ability of HIV-1 to infect target cells, we performed in vitro infection assays using HIV-1_YU2_ pseudoviruses carrying the observed mutations in gp120. Equivalent amounts of p24 (40 pg) were added to target cells for YU2_wt_ and the YU2 variant pseudoviruses, and luciferase expression was monitored in CD4^+^/CCR5^+^ TZM-bl cells as a measure of HIV-1 infectivity. Infection was greater for the wild-type YU2 virus than for the variants (Fig. 5*B*); three of the mutant viruses (YU2_G366E_, YU2_E466K_, and YU2_G471R_) were 3.8- to 6.6-fold less infectious than YU2_wt_, while a 24-fold reduction in viral entry fitness was observed for the YU2_G458D_ variant. The decreased infectivity of the mutant viruses could be related to changes affecting their binding to CD4, particularly for the YU2_G458D_ variant.

In vitro neutralization assays were performed to determine whether the mutations confer resistance to CD4-VLPs and CD4-CCR5-VLPs. All mutant viruses were ∼30-fold less sensitive than YU2_wt_ to sCD4 and CD4-Ig (Fig. 5*C* and *SI Appendix*, Table S5). Surprisingly, the mutant viruses were neutralized by CD4-VLPs and CD4-CCR5-VLPs at equivalent concentrations to those required for neutralization of the wild-type virus. This suggests that the mutations provided a selective advantage when exposed to short-lived, subneutralizing concentrations of CD4-CCR5-VLPs but did not decrease viral sensitivity to neutralizing CD4-VLP or CD4-CCR5-VLP concentrations. This demonstrates that CD4bs mutations that enable viral escape against conventional CD4-based inhibitors would not confer resistance to CD4-VLPs.

**Fig. 6.**
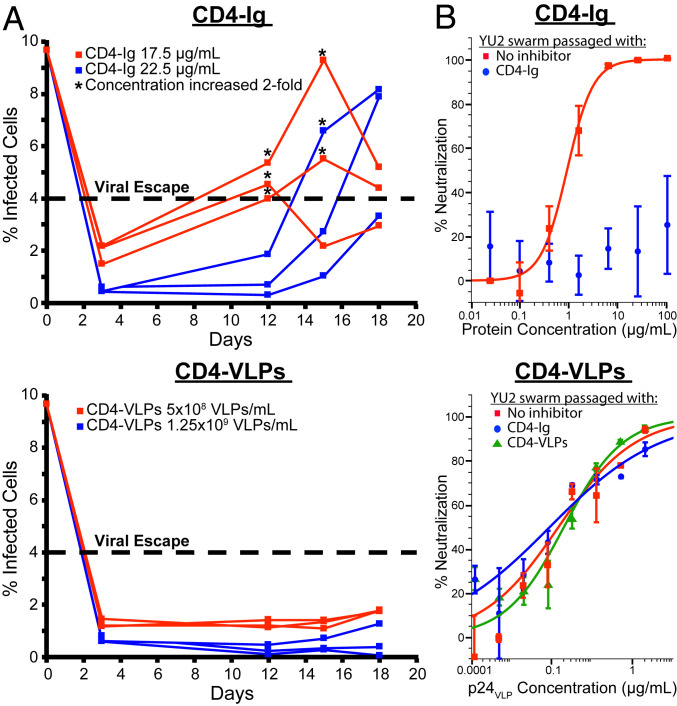
CD4-VLPs suppress HIV-1 replication and prevent viral escape in vitro. (*A*) In vitro evolution experiment comparing the ability of CD4-Ig and CD4-VLPs to suppress HIV-1 replication. Before the experiment, a diversified viral swarm had been generated by passaging replication-competent HIV-1_YU2_ on Rev-A3R5 CD4^+^ T cells for 21 d. On day 0, the viral supernatant was distributed into multiple wells, and IC_80_ (red) and IC_95_ (blue) concentrations of CD4-Ig (*Top*) or CD4-VLPs (*Bottom*) were added. After 1 h, fresh Rev-A3R5 cells were added, and infection rates were determined by measuring HIV-1–induced GFP expression by flow cytometry after 72 h. This cycle was repeated every 3 d, and a total of six cycles were completed. An infection rate >4% was set as a threshold to indicate viral escape (dashed line), and inhibitor concentrations were doubled (*) for replicates that surpassed this threshold for the next cycle. (*B*) In vitro neutralization assays for CD4-Ig (*Top*) and CD4-VLPs (*Bottom*) against HIV-1_YU2_ swarms that had been extensively passaged in the presence of no inhibitor (red), CD4-Ig (blue), or CD4-VLPs (green) (*Methods*). Inhibitor concentrations are shown as protein concentrations for CD4-Ig and as p24 concentrations for CD4-VLPs (Fig. 2*C* and *Methods*). Data points are presented as the mean ± SD of duplicate measurements.

To investigate whether HIV-1 is able to escape when exposed to neutralizing CD4-VLP concentrations, we performed in vitro evolution experiments. Replication-competent HIV-1_YU2_ was propagated on the Rev-A3R5 CD4^+^ T cell reporter line (35) for 21 d to generate a diversified viral population. Infection rates were maintained at ∼10% of infected cells by transferring the viral supernatant onto fresh target cells every 3 d. To compare the ability of CD4-Ig and CD4-VLPs to suppress viral replication, the viral swarm was distributed into multiple wells and exposed to their respective IC_80_s and IC_95_s for 1 h before fresh target cells were added. This cycle was repeated every 3 d, and a total of six cycles were completed. On day 3, HIV-1–induced GFP expression in Rev-A3R5 cells was measured by flow cytometry, which demonstrated that both inhibitors suppressed infection rates effectively (Fig. 6*A*). After four cycles (day 12), infection rates increased to >4% in the presence of 17.5 μg/mL CD4-Ig (IC_80_) for all replicates, which was set as a threshold to indicate viral escape. CD4-Ig concentrations were doubled to 35 μg/mL for the next cycle to assess whether the viral swarms were still sensitive to higher inhibitor concentrations. No signs of viral escape were observed for all other conditions at this point. After cycles 5 and 6 (days 15 and 18), infection rates also increased in the presence of 22.5 μg/mL CD4-Ig (IC_95_), and viral escape was observed for two of three replicates (Fig. 6*A*). Interestingly, infection rates for CD4-Ig (IC_80_) remained above 4% for two of three replicates even after the concentration was increased to 70 μg/mL after the fifth cycle. Infection rates remained low for CD4-VLPs at both concentrations, suggesting that a therapeutic that presents clusters of CD4 is more effective than traditional CD4-based inhibitors in controlling HIV-1 replication and preventing viral escape.

A modified version of a recently published in vitro evolution protocol (36) was used to evaluate whether HIV-1 can escape when intermittently exposed to increasing CD4-VLP concentrations. As above, the diversified HIV-1_YU2_ swarm was exposed to CD4-Ig and CD4-VLPs at starting concentrations of 17.5 μg/mL and 5 × 10^8^ VLPs/mL, respectively, for 1 h, followed by the addition of fresh target cells. After 3 d, infection rates were assessed under a fluorescent microscope, and the cycle was repeated in the absence of inhibitor to enable HIV-1 replication and ensure sufficient viral titers for the next selection cycle. This 6-d on/off cycle was repeated 15 times (90 d), and inhibitor concentrations were gradually increased to final concentrations of 280 μg/mL of CD4-Ig and 1.6 × 10^10^ CD4-VLPs/mL. To evaluate whether the selected viral swarms were resistant to the inhibitors, in vitro neutralization assays were performed. The diversified HIV-1_YU2_ control swarm that was continuously passaged in the absence of any inhibitor remained as sensitive as YU2_wt_ to CD4-Ig (IC_50_ = 0.91 μg/mL) and CD4-VLPs (IC_50_ = 0.02 μg p24/mL) (Fig. 6*B*); however, the HIV-1_YU2_ swarm that was intermittently exposed to increasing CD4-Ig concentrations was completely resistant to CD4-Ig (IC_50_ >100 μg/mL). In contrast, the viral swarm that was passaged in the presence of CD4-VLPs remained as sensitive to CD4-VLPs as the control swarm (IC_50_ = 0.014 μg p24/mL) (Fig. 6*B*). Importantly, CD4-VLPs also potently neutralized the CD4-Ig–resistant swarm (IC_50_ = 0.009 μg p24/mL), confirming that escape pathways against conventional CD4-based inhibitors are ineffective against CD4-VLPs. These results demonstrate that decoy therapeutics designed to present clusters of CD4 have the potential to effectively control HIV-1 replication and prevent viral escape.

## Discussion

Here we show that virus-like nanoparticles that mimic HIV-1 target cells by presenting an array of CD4 molecules neutralize HIV-1 with enhanced potency and breadth compared with conventional CD4-based inhibitors and bNAbs. In vivo studies in HIV-1_YU2_–infected hu-mice showed that intermittent subneutralizing CD4-CCR5-VLP plasma concentrations induced recurring CD4bs mutations in Env that reduced viral fitness and neutralization sensitivity to sCD4 and CD4-Ig in vitro, but all mutant viruses remained as sensitive as wild-type virus to CD4-VLPs at neutralizing concentrations. In vitro evolution studies demonstrated that CD4-VLPs effectively controlled HIV-1 replication, and viral escape was not observed. These results provide an explanation for the lack of efficacy of conventional CD4-based inhibitors and motivate the development of therapeutic strategies that more accurately mimic HIV-1 target cells to prevent viral escape and provide sustained suppression of HIV-1.

The enhanced neutralization potency and breadth of CD4-VLPs implies that membrane-associated display of multiple CD4 molecules is a more accurate mimic of the HIV-1 target cell compared with monovalent or bivalent CD4-based inhibitors. Cell surface CD4 receptors colocalize in nanoclusters that contain ∼4 to 15 molecules of CD4 (37, 38), which would facilitate multivalent binding to HIV-1 Envs. Because Env spikes are trimeric, each Env can bind up to three CD4 receptors, and multiple Envs may be engaged during the cell entry process (39, 40), leading to avidity effects. Compared with soluble CD4-based therapeutics, such as sCD4 and CD4-Ig, CD4-VLPs required >12,000-fold fewer copies of CD4 to neutralize a diverse panel of HIV-1 strains. This suggests that multiple CD4–Env interactions between CD4-VLPs and HIV-1 virions were formed, making it nearly impossible for HIV-1 to dissociate from CD4-VLPs and thereby minimizing the number of VLPs required to neutralize HIV-1. Cryo-ET imaging of CD4-VLPs is consistent with the involvement of distinct CD4 nanoclusters on a single VLP in neutralizing multiple virions simultaneously. Moreover, a single HIV-1 virion could be completely neutralized by two to four CD4-VLPs, as bound VLPs would sterically hinder the virus from interacting with target cells. The relatively large sizes of CD4-VLPs likely also prevented the enhancement of HIV-1 infection of target cells in vitro, which has been observed for sCD4 at low concentrations (41).

Twice-daily IP injections of large doses of CD4-CCR5-VLPs produced only intermittent subneutralizing plasma concentrations in hu-mice, likely due to rapid clearance by hepatic sinusoidal endothelial cells, which have been shown to clear nanoparticles at a rate of up to 10^8^ particles/min (31). As for conventional CD4-based inhibitors, interactions with class II major histocompatibility complex (MHC) proteins presented on antigen-presenting cells could shorten the half-life of CD4-CCR5-VLPs. Although human antigen-presenting cells have been shown to be generated in the hu-mouse model used in this study (42, 43), the bioavailabilities of control VLPs and CD4-CCR5-VLPs were similarly poor, suggesting that low VLP concentrations were not related to binding to human class II MHC-expressing cells. Despite poor bioavailability, intermittent subneutralizing CD4-CCR5-VLP concentrations elicited recurrent mutations in the CD4bs in circulating viruses. In vitro neutralization assays showed that YU2 viruses with these mutations were as sensitive to CD4-VLPs and CD4-CCR5-VLPs as the wild-type YU2 virus. We postulate that the mutations provided an advantage in the presence of short-lived subneutralizing CD4-CCR5-VLP concentrations, but did not confer resistance to neutralizing concentrations of CD4-VLPs and CD4-CCR5-VLPs. It is possible that the mutations protected the virus during short periods immediately after injections when there were relatively high plasma CD4-CCR5-VLP concentrations and/or protected the virus against constant exposure to low CD4-CCR5-VLP concentrations. In contrast, all mutant viruses were 30-fold less sensitive to sCD4 and CD4-Ig. For three of the four mutations, this loss of neutralization sensitivity was accompanied by only a fourfold to sevenfold reduction in infectivity. These results confirm that the ability of Env to bind multiple CD4 receptors on the target cell with avidity provides an escape route for HIV-1 against sCD4 and CD4-Ig, as the virus is able to tolerate CD4bs mutations that lower the intrinsic binding affinity for monomeric sCD4 or bivalent CD4-Ig without considerable fitness cost.

Our in vitro evolution experiments show that CD4-VLPs effectively suppressed viral replication at neutralizing concentrations, and that intermittent exposure to increasing CD4-VLP concentrations failed to select resistant viral swarms. This evolution strategy has been shown to generate viral populations completely resistant to CD4-Ig and the CD4bs bNAb NIH45-46 (36). A modest loss in viral sensitivity was also observed for the potent antibody-like inhibitor eCD4-Ig, a fusion of CD4-Ig and a CCR5-mimetic sulfopeptide that protected rhesus macaques from simian-HIV challenge following delivery using an adeno-associated virus vector (44). Similar to eCD4-Ig, viral swarms resistant to CD4-Ig remained sensitive to CD4-VLPs, demonstrating that escape pathways that are effective against conventional CD4-based inhibitors do not enable HIV-1 to escape against therapeutics that more accurately mimic HIV-1 target cells. Overall, our results suggest that effective viral escape against CD4-VLPs is difficult as the neutralization sensitivity to the therapeutic and the ability to infect target cells decrease concomitantly, thus forcing HIV-1 to become progressively less infectious.

CD4-VLPs were >100-fold more potent against a globally representative virus panel than the CD4bs bNAb 3BNC117 when comparing the required numbers of CD4 molecules vs. IgG Fabs. Importantly, CD4-VLPs were >12,000-fold more potent against three viral strains that were poorly neutralized by 3BNC117 and also potently neutralized two clinical viral isolates that were partially or completely resistant to 3BNC117 and other bNAbs. Therapeutics that present multiple copies of CD4 tethered to a surface have two potential advantages over bNAbs that could promote greater neutralization breadth and resistance to viral escape: (i) anti-Env IgGs (and CD4-Ig) are unlikely to use avidity effects to bind HIV-1 Env, because the low spike density on the viral surface and the distribution of epitopes on the Env trimer result in primarily monovalent binding that is vulnerable to escape through mutation of HIV-1 Env (16, 17); and (ii) although HIV-1 can mutate to become resistant to any single antibody (5), it must retain the ability to interact with its receptor to infect cells.

In summary, our results demonstrate that nanoparticles that mimic HIV-1 target cells by presenting multiple copies of membrane-associated CD4 neutralize HIV-1 with enhanced potency, breadth, and resistance to viral escape compared with conventional CD4-based inhibitors and CD4bs bNAbs. Therefore, we postulate that therapeutics that mimic viral target cells could prevent escape and permanently control HIV-1 infection by exposing a universal vulnerability—the requirement to bind clusters of CD4 on a target cell—that is potentially inherent to all HIV-1 strains and variants. Since direct injections of CD4-VLPs failed to achieve therapeutic concentrations in vivo, alternative therapeutic and/or delivery strategies that ensure durable bioavailability and minimize the requirement for repeated administrations need to be developed to translate this concept into a clinically feasible functional cure therapy.

## Materials and Methods

### VLP Production.

VLPs were produced by transiently transfecting Expi293 cells (Life Technologies) grown in Expi293 expression media (Life Technologies) on an orbital shaker at 37 °C and 8% CO_2_. Cells were transfected with a plasmid vector expressing Rev-independent HIV-1 Gag-Pol (pHDM-Hgpm2 plasmid; PlasmID Repository, Harvard Medical School) or a Gag-EGFP fusion protein (HIV-1 HXB2 Gag-EGFP expression vector; NIH AIDS Reagent Program). To generate CD4-VLPs and CD4-CCR5-VLPs, cells were cotransfected with a second plasmid (cDNA sequences of CD4 and CCR5 subcloned into the pHAGE-CMV-IRES-ZsGreen plasmid; PlasmID Repository, Harvard Medical School) encoding CD4 alone or CD4 and CCR5 at a DNA ratio of 4:1 Gag-Pol:CD4-(CCR5). Control VLPs were generated by expression of HIV-1 Gag-Pol alone. Expi293 cells were also transfected with CD4 in the absence of Gag-Pol to make CD4^+^ extracellular vesicles. At 48 to 72 h posttransfection, cells were centrifuged at 350 × *g* for 8 min, and supernatants were collected and passed through a 0.45-μm syringe filter.

### VLP Purification.

For in vitro neutralization experiments, VLPs were concentrated and buffer-exchanged into TZM-bl cell culture medium in Amicon Ultra-15 centrifugal filter units with a 100-kDa molecular weight cutoff (Millipore). For initial experiments, VLPs were isolated by ultracentrifugation at 28,000 rpm (96,000 × *g*) for 2 h at 4 °C using a SW32 Ti rotor and a Beckman L8-80M ultracentrifuge (Beckman Coulter) on a 20% wt/vol sucrose cushion. The supernatant was carefully aspirated, and the pellet was resuspended in 500 μL of culture medium at 4 °C overnight.

For quantitative Western blot analysis and cryo-ET imaging studies, 50 mL of filtered supernatant (combined from five independent CD4-VLP productions) was concentrated and buffer-exchanged into 500 μL of PBS by sucrose cushion ultracentrifugation as described above, centrifuged at 10,000 × *g* for 15 min, passed through a 0.45-μm syringe filter, and further purified by size exclusion chromatography on a Superose 6 10/300 column (GE Healthcare) equilibrated with 20 mM NaHPO_4_ (pH 7.4) and 150 mM NaCl. Fractions were collected and loaded onto 4 to 20% polyacrylamide gels (Bio-Rad) and stained with InstantBlue protein stain (Expedeon).

For in vivo experiments, control and CD4-CCR5-VLPs were concentrated from 1,000 mL to 5 mL in Slide-A-Lyzer dialysis cassettes (Thermo Fisher Scientific) that were immersed in a 40% wt/vol PEG (20 kDa) in ultrapure water concentrating solution (Thermo Fisher Scientific) and then buffer-exchanged into PBS.

### VLP Quantification.

VLP concentrations were quantified using a lentivirus-associated p24 ELISA kit (Cell Biolabs). To ensure accurate quantification of VLPs for in vitro neutralization studies, we used Gag-Pol instead of Gag-EGFP for generating VLPs, because detection of the Gag-encoded capsid protein p24 is less efficient for immature Gag than for mature Gag that had been processed by the HIV-1 protease encoded within Pol after budding (45). VLP concentrations were calculated using the following equation in accordance with the manufacturer’s directions: 1 ng p24 = 1.25 × 10^7^ VLPs (46, 47), which assumes that each VLP contains 2,000 molecules of p24 (20, 21).

### In Vitro Neutralization Assays.

The ability of VLPs to neutralize HIV-1 was evaluated using a pseudovirus-based TZM-bl assay (22). Pseudoviruses with Envs from YU2_wt_, YU variants, and strains from a 12-strain global HIV-1 panel (26) were generated in HEK293T cells as described previously (48). Serial dilutions of control VLPs, CD4-VLPs, and CD4-CCR5-VLPs were incubated with pseudovirus for 1 h at 37 °C. TZM-bl cells (NIH AIDS Reagents Program) that express a Tat-inducible luciferase reporter gene were added, and luminescence was measured after 48 h. The HIV-1 neutralization activity of CD4-VLPs was compared with that of sCD4, CD4-Ig, and the CD4bs bNAb 3BNC117. Neutralization assays were also performed against primary virus isolates obtained from the latent reservoirs of two HIV-1–infected patients who received repeated infusions of 3BNC117 (27). Viruses were isolated from peripheral blood mononuclear cells by a quantitative and qualitative viral outgrowth assay (Q^2^VOA) as described previously (49).

The number of CD4 molecules/mL required to achieve 50% neutralization was calculated by multiplying the VLP concentration (derived from the p24 ELISA) at the IC_50_ (derived from an in vitro neutralization assay) by the average number of CD4 copies per VLP (derived from quantitative Western blot analysis). For sCD4 D1D2 (26 kDa), CD4-Ig (100 kDa), and 3BNC117 (150 kDa), the numbers of CD4 molecules/mL (sCD4, CD4-Ig) or Fab molecules/mL (3BNC117) were calculated by converting the measured IC_50_ values (in μg/mL) to molar concentrations. The respective numbers of inhibitor molecules were then derived from molar IC_50_ concentrations using Avogadro’s number. The numbers of inhibitor molecules (CD4 or Fab) were then multiplied by a factor of one (sCD4) or two (CD4-Ig, 3BNC117), depending on whether the inhibitor molecule contained one or two CD4/Fab copies. IC_50_ values calculated from independent assays generally agreed to within twofold to fourfold (*SI Appendix*, Fig. S3).

### In Vivo Studies.

Studies in hu-mice were performed in accordance with the recommendations in the NIH’s *Guide for the Care and Use of Laboratory Animals*. The protocol was reviewed and approved by The Rockefeller University’s Institutional Animal Care and Use Committee, and experiments were designed in accordance with established guidelines at The Rockefeller University (protocol no. 13618-H).

Hu-mice were generated as described previously (50). In brief, human CD34^+^ hematopoietic stem cells were obtained from human fetal livers (Human Fetal Tissue Repository) and injected intrahepatically into irradiated nonobese diabetic Rag1^−/−^ IL2rg^null^ (NOD.Cg-Rag1tm1Mom Il2rgtm1Wjl/SzJ) mice (The Jackson Laboratory). Half-life studies for VLPs were performed in uninfected hu-mice. CD4-CCR5-VLPs (610 ng of p24) were injected IP into five hu-mice, and a single blood sample was taken from each animal after 20 min, 1 h, 2 h, 4 h, or 6 h. Plasma CD4-CCR5-VLP concentrations were measured by lentivirus-associated p24 ELISA (Cell Biolabs).

For treatment experiments, hu-mice were infected with HIV-1_YU2_, and viral plasma loads were measured by qRT-PCR at 10 d postinfection as described previously (50). Infected hu-mice were distributed into four treatment groups: no treatment (group I), twice-daily IP injections of 480 ng p24 of control VLPs (group II) or CD4-CCR5-VLPs (group III), or twice-weekly administration of 1 mg of the bNAb 10–1074 (group IV). Treatments were continued for a total of 10 d, and viral plasma loads were measured on days 3, 6, and 10 by qRT-PCR as described previously (50).

### Viral Fitness Assay.

To evaluate the ability of HIV-1_YU2_ Env mutants to enter and infect target cells, a previously described infection assay (51, 52) was used with minor modifications. Here 40 pg p24 of YU2_wt_ or mutant YU2 pseudoviruses (quantified by lentivirus-associated p24 ELISA; Cell Biolabs) were added to TZM-bl reporter cells in the presence of 30 μg/mL DEAE-dextran. After a 48-h incubation at 37 °C, cells were lysed, and luminescence was measured after the addition of britelite plus (PerkinElmer). The average luminescence among eight 8 replicates was calculated for YU2_wt_ and each YU2 variant, and the experiment was repeated three times with different pseudovirus batches. The viral entry fitness of the YU2 mutant viruses was calculated as a function of the reduction in average luminescence compared with YU2_wt_.

### In Vitro Evolution Assays.

Replication-competent HIV-1_NL4-3_ carrying the HIV-1_YU2_ envelope (53) was passaged on Rev-A3R5 CD4^+^ T cells (35) (Cube BioSystems) for 21 d to diversify the viral population. Rev-A3R5 cells were maintained in RPMI 1640 medium supplemented with 10% FBS, 1% pen-strep, 1% l-glutamine, 1 mg/mL geneticin, and 1 μg/mL puromycin at 37 °C and 5% CO_2_. To test whether CD4-VLPs can suppress viral replication, 30 μL of viral supernatant was added to multiple wells on a 48-well plate. Previously determined IC_80_s and IC_95_s of CD4-Ig (17.5 and 22.5 μg/mL) and CD4-VLPs (5 × 10^8^ and 1.25 × 10^9^ VLPs/mL) or no inhibitor were added to the wells in triplicate in the presence of 5 μg/mL DEAE-dextran, and then media was added for a final volume of 500 μL. After 1 h of incubation at 37 °C, 5 × 10^4^ Rev-A3R5 cells were added, and the plates were incubated for 16 h at 37 °C. The next day, cells were centrifuged at 350 × *g* for 8 min, supernatants were removed, and cells were resuspended in 500 μL of fresh medium. After 48 h, infection rates were quantified by measuring HIV-1–induced GFP expression in Rev-A3R5 cells by flow cytometry (MACSQuant; Miltenyi Biotec). For the second cycle, 350 μL of viral supernatants for each condition were transferred into fresh 48-well plates and inhibitors, DEAD-dextran, and cells were added as in cycle 1. A total of six cycles were performed, and infection rates were determined after cycles 4 to 6. Infection rates in the absence of inhibitor were maintained at ∼10% infected cells, and to account for variations between cycles, infection rates were normalized to a viral control infection of 10%. Viral escape was defined as >4% infected cells (60% neutralization), and inhibitor concentrations were increased twofold for replicates that surpassed this threshold for the next cycle.

A modified version of a previously described in vitro evolution protocol (36) was used to evaluate whether HIV-1 can escape from CD4-VLPs when intermittently exposed to increasing inhibitor concentrations. Here 10 to 20 μL of viral supernatant was added to multiple wells on a 96-well plate, then CD4-Ig and CD4-VLPs were added at starting concentrations of 17.5 μg/mL and 5 × 10^8^ VLPs/mL, respectively, in the presence of 5 μg/mL DEAE-dextran, and finally medium was added for a final volume of 200 μL. After a 1-h incubation at 37 °C, 2.5 × 10^4^ cells were added, and the plates were incubated at 37 °C. After 8 h, cells were centrifuged, supernatants were removed, and cells were resuspended in 200 μL of fresh medium. After 64 h, infection rates were assessed using a fluorescent microscope (Zeiss AX10). The second cycle was performed in the absence of inhibitor to enable HIV-1 replication and ensure sufficient viral titers for the next selection cycle. This 6-d on/off cycle was repeated 15 times (90 d), and inhibitor concentrations were doubled every two to four cycles to final concentrations of 280 μg/mL of CD4-Ig and 1.6 × 10^10^ CD4-VLPs/mL. To maintain infections at higher inhibitor concentrations, up to 150 μL of viral supernatant was passaged, and repeated cycles in the absence of inhibitor were performed. After the final cycle, viral supernatants for each condition (no inhibitor, CD4-Ig, and CD4-VLPs) were collected and TZMbl assays were performed as described above.

### Statistical Analysis.

Concentrations at which half-maximal neutralization was observed (IC_50_ values) were calculated using software in the HIV Antibody Database (54). The levels of conservation of the mutated residues and the respective substitutions observed in *env* sequences obtained from HIV-1–infected and CD4-CCR5-VLP–treated hu-mice were determined through filtered web alignment of HIV-1 sequences in the Los Alamos National Laboratory HIV Database (https://www.hiv.lanl.gov/).

### Data and Materials Availability.

All data associated with this study are available in the main text or *SI Appendix*.

## Supplementary Material

Supplementary File

Supplementary File

## References

[1] Choudhary S. K., Margolis D. M., Curing HIV: Pharmacologic approaches to target HIV-1 latency. Annu. Rev. Pharmacol. Toxicol. 51, 397–418 (2011). 21210747 10.1146/annurev-pharmtox-010510-100237 PMC3958947

[2] Bruel T., Schwartz O., Markers of the HIV-1 reservoir: Facts and controversies. Curr. Opin. HIV AIDS 13, 383–388 (2018). 29846244 10.1097/COH.0000000000000482

[3] Siliciano R. F., Greene W. C., HIV latency. Cold Spring Harb. Perspect. Med. 1, a007096 (2011). 22229121 10.1101/cshperspect.a007096 PMC3234450

[4] Katlama C.., Barriers to a cure for HIV: New ways to target and eradicate HIV-1 reservoirs. Lancet 381, 2109–2117 (2013). 23541541 10.1016/S0140-6736(13)60104-X PMC3815451

[5] Gardner M. R., Farzan M., Engineering antibody-like inhibitors to prevent and treat HIV-1 infection. Curr. Opin. HIV AIDS 12, 294–301 (2017). 28422793 10.1097/COH.0000000000000367 PMC5389584

[6] Falkenhagen A., Joshi S., Further characterization of the bifunctional HIV entry inhibitor sCD4-FI_T45_. Mol. Ther. Nucleic Acids 7, 387–395 (2017). 28624214 10.1016/j.omtn.2017.04.017 PMC5432676

[7] Harrison S. C., Viral membrane fusion. Virology 479–480, 498–507 (2015). 10.1016/j.virol.2015.03.043 PMC4424100 25866377

[8] Schooley R. T.., Recombinant soluble CD4 therapy in patients with the acquired immunodeficiency syndrome (AIDS) and AIDS-related complex. A phase I-II escalating dosage trial. Ann. Intern. Med. 112, 247–253 (1990). 2297203 10.7326/0003-4819-112-4-247

[9] Schacker T.., Phase I study of high-dose, intravenous rsCD4 in subjects with advanced HIV-1 infection. J. Acquir. Immune Defic. Syndr. Hum. Retrovirol. 9, 145–152 (1995). 7749791

[10] Daar E. S., Li X. L., Moudgil T., Ho D. D., High concentrations of recombinant soluble CD4 are required to neutralize primary human immunodeficiency virus type 1 isolates. Proc. Natl. Acad. Sci. U.S.A. 87, 6574–6578 (1990). 2395859 10.1073/pnas.87.17.6574 PMC54579

[11] Klasse P. J., McKeating J. A., Soluble CD4 and CD4 immunoglobulin-selected HIV-1 variants: A phenotypic characterization. AIDS Res. Hum. Retroviruses 9, 595–604 (1993). 8369164 10.1089/aid.1993.9.595

[12] McKeating J. A.., Resistance of a human serum-selected human immunodeficiency virus type 1 escape mutant to neutralization by CD4 binding site monoclonal antibodies is conferred by a single amino acid change in gp120. J. Virol. 67, 5216–5225 (1993). 7688820 10.1128/jvi.67.9.5216-5225.1993 PMC237919

[13] Grupping K.., MiniCD4 protein resistance mutations affect binding to the HIV-1 gp120 CD4 binding site and decrease entry efficiency. Retrovirology 9, 36 (2012). 22551420 10.1186/1742-4690-9-36 PMC3408336

[14] Arthos J.., Biochemical and biological characterization of a dodecameric CD4-Ig fusion protein: Implications for therapeutic and vaccine strategies. J. Biol. Chem. 277, 11456–11464 (2002). 11805109 10.1074/jbc.M111191200

[15] Traunecker A., Schneider J., Kiefer H., Karjalainen K., Highly efficient neutralization of HIV with recombinant CD4-immunoglobulin molecules. Nature 339, 68–70 (1989). 2541344 10.1038/339068a0

[16] Klein J. S., Bjorkman P. J., Few and far between: How HIV may be evading antibody avidity. PLoS Pathog. 6, e1000908 (2010). 20523901 10.1371/journal.ppat.1000908 PMC2877745

[17] Galimidi R. P.., Intra-spike crosslinking overcomes antibody evasion by HIV-1. Cell 160, 433–446 (2015). 25635457 10.1016/j.cell.2015.01.016 PMC4401576

[18] Amitai A., Chakraborty A. K., Kardar M., The low spike density of HIV may have evolved because of the effects of T helper cell depletion on affinity maturation. PLoS Comput. Biol. 14, e1006408 (2018). 30161121 10.1371/journal.pcbi.1006408 PMC6150518

[19] Gheysen D.., Assembly and release of HIV-1 precursor Pr55gag virus-like particles from recombinant baculovirus-infected insect cells. Cell 59, 103–112 (1989). 2676191 10.1016/0092-8674(89)90873-8

[20] Carlson L. A.., Three-dimensional analysis of budding sites and released virus suggests a revised model for HIV-1 morphogenesis. Cell Host Microbe 4, 592–599 (2008). 19064259 10.1016/j.chom.2008.10.013 PMC3454483

[21] Goicochea N. L.., Structure and stoichiometry of template-directed recombinant HIV-1 Gag particles. J. Mol. Biol. 410, 667–680 (2011). 21762807 10.1016/j.jmb.2011.04.012 PMC3140650

[22] Montefiori D. C., “Evaluating neutralizing antibodies against HIV, SIV, and SHIV in luciferase reporter gene assays” in Curr. Protoc. Immunol., (2005), Vol. Chapter 12, p. Unit 12.11. 10.1002/0471142735.im1211s64 18432938

[23] Tkach M., Théry C., Communication by extracellular vesicles: Where we are and where we need to go. Cell 164, 1226–1232 (2016). 26967288 10.1016/j.cell.2016.01.043

[24] Caskey M.., Viraemia suppressed in HIV-1-infected humans by broadly neutralizing antibody 3BNC117. Nature 522, 487–491 (2015). 25855300 10.1038/nature14411 PMC4890714

[25] Mendoza P.., Combination therapy with anti-HIV-1 antibodies maintains viral suppression. Nature 561, 479–484 (2018). 30258136 10.1038/s41586-018-0531-2 PMC6166473

[26] deCamp A.., Global panel of HIV-1 Env reference strains for standardized assessments of vaccine-elicited neutralizing antibodies. J. Virol. 88, 2489–2507 (2014). 24352443 10.1128/JVI.02853-13 PMC3958090

[27] Cohen Y. Z.., Relationship between latent and rebound viruses in a clinical trial of anti-HIV-1 antibody 3BNC117. J. Exp. Med. 215, 2311–2324 (2018). 30072495 10.1084/jem.20180936 PMC6122972

[28] Kolchinsky P.., Adaptation of a CCR5-using, primary human immunodeficiency virus type 1 isolate for CD4-independent replication. J. Virol. 73, 8120–8126 (1999). 10482561 10.1128/jvi.73.10.8120-8126.1999 PMC112828

[29] Gorry P. R.., Increased CCR5 affinity and reduced CCR5/CD4 dependence of a neurovirulent primary human immunodeficiency virus type 1 isolate. J. Virol. 76, 6277–6292 (2002). 12021361 10.1128/JVI.76.12.6277-6292.2002 PMC136234

[30] Laurén A., Vincic E., Hoshino H., Thorstensson R., Fenyö E. M., CD4-independent use of the CCR5 receptor by sequential primary SIVsm isolates. Retrovirology 4, 50 (2007). 17645788 10.1186/1742-4690-4-50 PMC1950888

[31] Mates J. M.., Mouse liver sinusoidal endothelium eliminates HIV-like particles from blood at a rate of 100 million per minute by a second-order kinetic process. Front. Immunol. 8, 35 (2017). 28167948 10.3389/fimmu.2017.00035 PMC5256111

[32] Caskey M.., Antibody 10-1074 suppresses viremia in HIV-1-infected individuals. Nat. Med. 23, 185–191 (2017). 28092665 10.1038/nm.4268 PMC5467219

[33] Dreja H., Pade C., Chen L., McKnight Á., CD4 binding site broadly neutralizing antibody selection of HIV-1 escape mutants. J. Gen. Virol. 96, 1899–1905 (2015). 25762593 10.1099/vir.0.000120 PMC4835949

[34] Lynch R. M.., HIV-1 fitness cost associated with escape from the VRC01 class of CD4 binding site neutralizing antibodies. J. Virol. 89, 4201–4213 (2015). 25631091 10.1128/JVI.03608-14 PMC4442379

[35] McLinden R. J.., Detection of HIV-1 neutralizing antibodies in a human CD4^+^/CXCR4^+^/CCR5^+^ T-lymphoblastoid cell assay system. PLoS One 8, e77756 (2013). 24312168 10.1371/journal.pone.0077756 PMC3842913

[36] Fellinger C. H.., eCD4-Ig limits HIV-1 escape more effectively than CD4-Ig or a broadly neutralizing antibody. J. Virol. 93, e00443-19 (2019). 31068428 10.1128/JVI.00443-19 PMC6600210

[37] Roh K. H., Lillemeier B. F., Wang F., Davis M. M., The coreceptor CD4 is expressed in distinct nanoclusters and does not colocalize with T-cell receptor and active protein tyrosine kinase p56lck. Proc. Natl. Acad. Sci. U.S.A. 112, E1604–E1613 (2015). 25829544 10.1073/pnas.1503532112 PMC4386407

[38] Singer I. I.., CCR5, CXCR4, and CD4 are clustered and closely apposed on microvilli of human macrophages and T cells. J. Virol. 75, 3779–3790 (2001). 11264367 10.1128/JVI.75.8.3779-3790.2001 PMC114869

[39] Sougrat R.., Electron tomography of the contact between T cells and SIV/HIV-1: Implications for viral entry. PLoS Pathog. 3, e63 (2007). 17480119 10.1371/journal.ppat.0030063 PMC1864992

[40] Chojnacki J.., Maturation-dependent HIV-1 surface protein redistribution revealed by fluorescence nanoscopy. Science 338, 524–528 (2012). 23112332 10.1126/science.1226359

[41] Sullivan N.., Determinants of human immunodeficiency virus type 1 envelope glycoprotein activation by soluble CD4 and monoclonal antibodies. J. Virol. 72, 6332–6338 (1998). 9658072 10.1128/jvi.72.8.6332-6338.1998 PMC109776

[42] Ishikawa F.., Development of functional human blood and immune systems in NOD/SCID/IL2 receptor gamma chain(null) mice. Blood 106, 1565–1573 (2005). 15920010 10.1182/blood-2005-02-0516 PMC1895228

[43] Saito Y., Ellegast J. M., Manz M. G., Generation of humanized mice for analysis of human dendritic cells. Methods Mol. Biol. 1423, 309–320 (2016). 27142026 10.1007/978-1-4939-3606-9_22

[44] Gardner M. R.., AAV-expressed eCD4-Ig provides durable protection from multiple SHIV challenges. Nature 519, 87–91 (2015). 25707797 10.1038/nature14264 PMC4352131

[45] Hammonds J.., Gp120 stability on HIV-1 virions and Gag-Env pseudovirions is enhanced by an uncleaved Gag core. Virology 314, 636–649 (2003). 14554091 10.1016/s0042-6822(03)00467-7

[46] Gach J. S.., Human immunodeficiency virus type-1 (HIV-1) evades antibody-dependent phagocytosis. PLoS Pathog. 13, e1006793 (2017). 29281723 10.1371/journal.ppat.1006793 PMC5760106

[47] Vink C. A.., Eliminating HIV-1 packaging sequences from lentiviral vector proviruses enhances safety and expedites gene transfer for gene therapy. Mol. Ther. 25, 1790–1804 (2017). 28550974 10.1016/j.ymthe.2017.04.028 PMC5542766

[48] Diskin R.., Increasing the potency and breadth of an HIV antibody by using structure-based rational design. Science 334, 1289–1293 (2011). 22033520 10.1126/science.1213782 PMC3232316

[49] Lorenzi J. C.., Paired quantitative and qualitative assessment of the replication-competent HIV-1 reservoir and comparison with integrated proviral DNA. Proc. Natl. Acad. Sci. U.S.A. 113, E7908–E7916 (2016). 27872306 10.1073/pnas.1617789113 PMC5150408

[50] Klein F.., HIV therapy by a combination of broadly neutralizing antibodies in humanized mice. Nature 492, 118–122 (2012). 23103874 10.1038/nature11604 PMC3809838

[51] Brandenberg O. F.., Partial rescue of V1V2 mutant infectivity by HIV-1 cell-cell transmission supports the domain’s exceptional capacity for sequence variation. Retrovirology 11, 75 (2014). 25287422 10.1186/s12977-014-0075-y PMC4190450

[52] Li H.., Envelope residue 375 substitutions in simian-human immunodeficiency viruses enhance CD4 binding and replication in rhesus macaques. Proc. Natl. Acad. Sci. U.S.A. 113, E3413–E3422 (2016). 27247400 10.1073/pnas.1606636113 PMC4914158

[53] Zhang Y. J.., Envelope-dependent, cyclophilin-independent effects of glycosaminoglycans on human immunodeficiency virus type 1 attachment and infection. J. Virol. 76, 6332–6343 (2002). 12021366 10.1128/JVI.76.12.6332-6343.2002 PMC136233

[54] West A. P. Jr.., Computational analysis of anti-HIV-1 antibody neutralization panel data to identify potential functional epitope residues. Proc. Natl. Acad. Sci. U.S.A. 110, 10598–10603 (2013). 23754383 10.1073/pnas.1309215110 PMC3696754

