# Supplementary material for: Nanoparticles presenting clusters of CD4 expose a universal vulnerability of HIV-1 by mimicking target cells
Source: Proc Natl Acad Sci U S A. 2020 Jul 20;117(31):18719–28. doi: 10.1073/pnas.2010320117 (PMC7414181; doi:10.1073/pnas.2010320117)
Supplement: Supplementary File [file pnas.2010320117.sapp.pdf]

**SI Appendix**

Supplementary materials and methods

Fig. S1. Purification of CD4-VLPs for quantitative Western blot analysis and cryo-ET.

Fig. S2. Quantitative Western blot analysis.

Fig. S3. Comparison of neutralization by different CD4-VLP batches.

Fig. S4. Neutralization activity requires Gag and CD4.

Fig. S5. CD4-CCR5-VLP treatment selects mutations in HIV-$1_{YU2}$-infected humanized mice.

Fig. S6. SPR binding analysis of YU2 gp120 mutants.

Table S1. $IC_{50}$s for CD4-VLPs and CD4-CCR5-VLPs against a panel of HIV-1 strains.

Table S2. $IC_{50}$s for sCD4, CD4-Ig, and 3BNC117 against a panel of HIV-1 strains.

Table S3. $IC_{50}$s for CD4-VLPs and various bNAbs against HIV-1 isolates obtained from infected individuals.

Table S4. $K_D$, $k_a$, $k_d$ values for binding of YU2 gp120 variants to CD4-Ig.

Table S5. $IC_{50}$s for CD4-VLPs, CD4-CCR5-VLPs, sCD4, and CD4-Ig against $YU2_{wt}$ and YU2 mutant pseudoviruses.

Movie S1. Tomographic reconstruction of CD4-VLPs bound to BG505 SOSIP.664.

www.pnas.org/cgi/doi/10.1073/pnas.2010320117

## Supplementary materials and methods

### Protein expression

6 x His-tagged $YU2_{wt}$ and mutant gp120 proteins were expressed by transient transfection using pTT5 expression vectors (NRC Biotechnology Research Institute) in HEK293-6E cells (National Research Council of Canada). The cells were grown in FreeStyle 293 expression media (Gibco) on an orbital shaker at 37°C and 8% $CO_2$. gp120 proteins were purified from cell supernatants by Ni-NTA affinity chromatography (GE Healthcare) followed by size exclusion chromatography (SEC) using a Superdex 200 30/200 column (GE Healthcare). sCD4 (D1-D2 and D1-D4 constructs), CD4-Ig, HIV-1 bNAbs, control IgGs, and a soluble native-like BG505 SOSIP.664 Env trimer (1) were expressed and purified from transfected cell supernatants as described (2, 3). Proteins were stored at 4°C in 20 mM Tris, pH 8.0, and 150 mM sodium chloride (TBS buffer).

### Western blot analysis

The presence of CD4 and CCR5 on VLPs was confirmed by Western blots. Briefly, total protein concentrations for all samples were measured using the Pierce BCA protein assay kit (Thermo Fisher Scientific). 1 μg of total protein was separated by SDS-PAGE and transferred to nitrocellulose membranes (0.2 μm) (GE Healthcare). The following antibodies were used for detecting CD4 and CCR5: rabbit anti-CD4 (ab133616; Abcam) at 1:10,000, rat anti-CCR5 (ab111300; Abcam) at 1:2,000, HRP-conjugated mouse anti-rabbit IgG (211-032-171; Jackson ImmunoResearch) at 1:1,000, and HRP-conjugated mouse anti-rat IgG (3065-05; Southern Biotech) at 1:1,000. Protein bands were visualized using ECL Prime Western Blotting Detection Reagent (GE Healthcare).

The number of CD4 molecules displayed on the surface of CD4-VLPs was estimated by quantitative Western blot analysis. Various dilutions of SEC-purified CD4-VLPs and known amounts of purified Gag p55 (Abcam) and sCD4 D1-D4 were separated by gel electrophoresis and transferred to nitrocellulose membranes (GE Healthcare). Gag-EGFP was detected using a polyclonal rabbit anti-p17/p55 antibody (ab63195; Abcam) and HRP-conjugated mouse anti-rabbit IgG (211-032-171; Jackson ImmunoResearch) at dilutions of 1:2,500 and 1:1,000, respectively. CD4 was detected as described above. Band intensities of the Gag and sCD4 standards and CD4-VLP sample dilutions were measured using ImageJ to estimate the concentrations of Gag-EGFP and CD4. The number of CD4 copies per VLP was derived from the molar Gag-EGFP (84 kDa) to CD4 (48 kDa) ratio, assuming that each VLP contains 2,000 copies of Gag-EGFP (4, 5). The average number of CD4 copies per VLP was calculated using results from six quantitative Western blot replicate experiments.

**Cryo-ET**

SEC-purified CD4-VLPs were incubated with 10 µg/mL BG505 SOSIP.664 for 4 hours at room temperature. CD4-VLP/BG505 SOSIP.664 complexes were then re-purified over SEC. Cryo-EM grids containing CD4-VLP/BG505 SOSIP.664 complexes were prepared using a Mark IV Vitrobot (ThermoFisher Scientific) operated at 21°C and 100% humidity. 2.5 µL of sample was mixed with 1 µL of 10 nm colloidal gold beads (Sigma-Aldrich) and applied to 200 mesh Quantifoil R2/1 grids, blotted for 3.5 s, and then plunge-frozen in liquid ethane surrounded by liquid nitrogen.

Cryo-grids were loaded into a 300kV Titan Krios transmission electron microscope (ThermoFisher Scientific) equipped with a Gatan energy filter (slit width 20 eV)

operating at a nominal 42,000x magnification. Tilt series were recorded on a K3 direct electron detector (Gatan) in counting mode with a pixel size of 2.176 Å•pixel$^{-1}$ using SerialEM software (6). The defocus range was -2 to -5 µm with a total dose of ~80 $e^{-}$/Å$^{2}$ per tilt series. Tilt series images were collected from -60° to 60° at 3° intervals using dose-symmetric tilt scheme (7). Images were aligned and reconstructed using IMOD software (8).

**Surface plasmon resonance binding experiments**

SPR experiments were performed using a Biacore T200 instrument (GE Healthcare). Protein A was immobilized on a CM5 chip by primary amine chemistry (Biacore manual), and 10 nM CD4-Ig or a non-HIV-1 binding IgG (mG053) was injected for 60 seconds at a flow rate of 10 µl/min as described (9), resulting in attachment of 250-300 resonance units (RUs) of an Fc-containing protein. 1 µM human Fc was injected for 60 seconds at a flow rate of 10 µl/min to block remaining protein A sites, followed by a concentration series of YU2 gp120 variants (a total of six 4-fold dilutions starting from a top concentration of 250 nM) for a contact time of 60 seconds and a dissociation time of 300 sec at a flow rate of 30 µl/min. Binding reactions were allowed to reach equilibrium, and $K_D$s were calculated from the ratio of association and dissociation rates ($K_D = k_d/k_a$). Kinetic constants were derived using Biacore T200 Evaluation Software v3.2 by simultaneously fitting the association and dissociation phases of all curves in each data set using a 1:1 binding. Flow cells were regenerated with 1 M guanidine HCl and/or 10 mM glycine pH 2.0 at a flow rate of 90 µl/min.

**Single genome sequencing of HIV-1 *env***

HIV-1 *env* sequences from 2 hu-mice treated with control VLPs and 3 hu-mice treated with CD4-CCR5-VLPs were obtained as previously described (10). For single

genome sequencing of viruses, HIV-1 RNA was extracted from 200 uL plasma samples collected on day 10 of VLP treatment from each mouse (QIAGEN MinElute Virus Spin Kit), and cDNA was generated by reverse transcription (SuperScript III Reverse Transcriptase). Samples were treated with RNaseH to remove residual RNA for 20 min at 37°C. cDNA samples were diluted and amplified by two cycles of nested PCR with previously-described gp160-specific primers (11). PCR products with the expected size of HIV-1 *env* and amplification efficiencies of <30% were selected for library preparation using the Illumina Nextera DNA Sample Preparation Kit. Sequencing was performed as described (12).

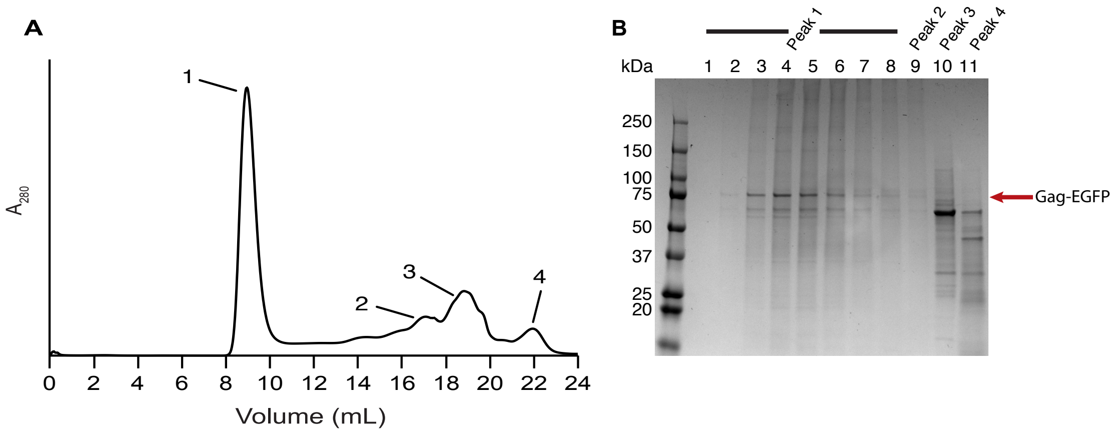


**Fig. S1. Purification of CD4-VLPs for quantitative Western blot analysis and cryo-ET.** (**A**) Size exclusion chromatogram of CD4-VLPs following purification by sucrose cushion ultracentrifugation. (**B**) SDS-PAGE analysis of fractions corresponding to peaks 1 (lanes 1-8), 2 (lane 9), 3 (lane 10), and 4 (lane 11). Gag-EGFP-containing fractions corresponding to lanes 3-6 were selected for quantitative Western blot analysis and cryo-ET imaging.

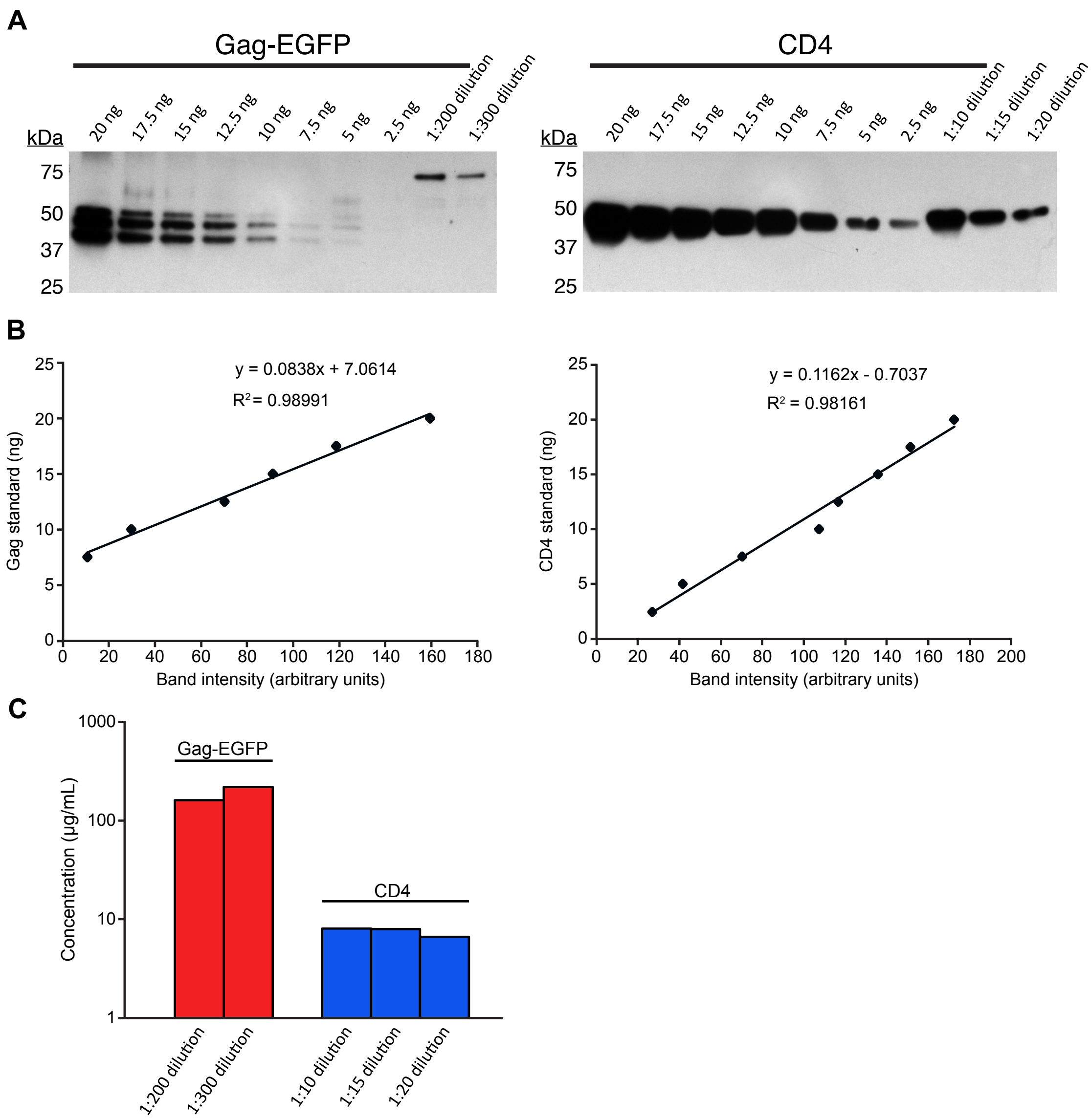


**Fig. S2. Quantitative Western blot analysis.** Supernatants from five independent CD4-VLP productions were combined and purified (Fig. S1). Representative Gag and CD4 blots from six quantitative Western blot replicate experiments are shown, from which an average of 140 +/- 48 CD4 copies per VLP was calculated (results ranged from 60-190 CD4 copies per VLP). (**A**) Western blots showing decreasing amounts of Gag (55 kDa) and sCD4 (43 kDa) standards, respectively (lanes 1-8). Two degradation bands were present for Gag standards, which result from proteolytic removal of p6 (49 kDa) and p7 (42 kDa). Various dilutions of the SEC-purified CD4-VLPs (Fig. S1) were included to determine the concentrations of Gag-EGFP (84 kDa) and CD4 (48 kDa). (**B**) Standard curves corresponding to measured

band intensities for Gag (left) and sCD4 (right) standards using linear regression analysis. Band intensities were measured using ImageJ and all three bands were included for the Gag standard. Linear regression equations and correlation coefficients are displayed. (**C**) Gag-EGFP and CD4 concentrations determined for each sample dilution using the linear regression equations from (B).

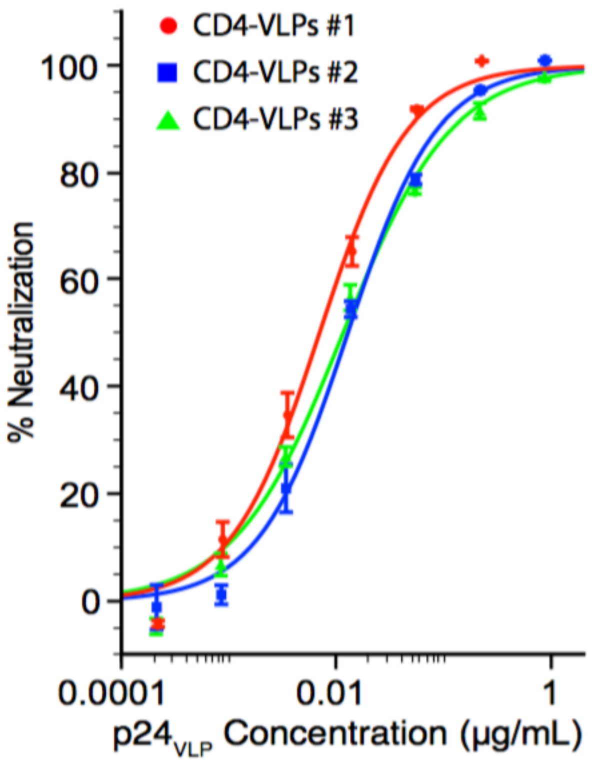


**Fig. S3. Comparison of neutralization by different CD4-VLP batches.** Neutralization curves for three different CD4-VLP batches against HIV-$1_{YU2}$. Data points are presented as the mean and standard deviation of duplicate measurements.

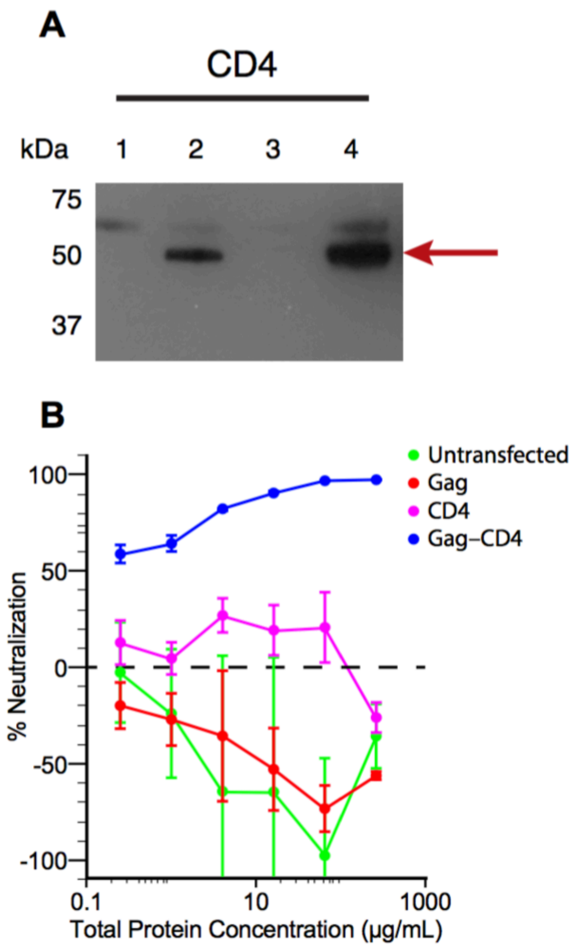


**Fig. S4. Neutralization activity requires Gag and CD4.** (**A**) Western blot analysis comparing relative CD4 levels in concentrated supernatants containing extracellular veciscles (EVs) only (Lane 1, untransfected control cells, and Lane 2, transfected cells expressing CD4) or EVs and VLPs (Lane 3, transfected cells expressing Gag, and Lane 4, transfected cells expressing Gag and CD4). (**B**) In vitro neutralization assay against HIV-$1_{YU2}$ pseudovirus comparing the neutralization activity of EVs and VLPs. Equivalent total protein concentrations were used for concentrated supernatants collected from transfected Expi293 cells under the following conditions: untransfected cells (EVs only), cells expressing Gag (EVs and VLPs, no CD4), cells expressing CD4 (CD4-positive EVs), and cells expressing Gag and CD4 (CD4-positive EVs and CD4-VLPs). Data points are presented as the mean and standard deviation of duplicate measurements.

| | $IC_{50}$ | | | | | |
|---|---|---|---|---|---|---|
| **HIV Strain** | **CD4-VLPs (µg p24/mL)** | **CD4-VLPs (VLPs/mL)** | **CD4-VLPs (CD4 copies/mL)** | **CD4-CCR5-VLPs (µg p24/mL)** | **CD4-CCR5-VLPs (VLPs/mL)** | **CD4-CCR5-VLPs (CD4 copies/mL)** |
| YU2 | 0.012 | $1.5 \times 10^8$ | $2.1 \times 10^{10}$ | 0.006 | $0.8 \times 10^8$ | $1.1 \times 10^{10}$ |
| CNE55 | 0.009 | $1.1 \times 10^8$ | $1.6 \times 10^{10}$ | 0.008 | $1.0 \times 10^8$ | $1.4 \times 10^{10}$ |
| Tro11 | 0.009 | $1.1 \times 10^8$ | $1.6 \times 10^{10}$ | 0.010 | $1.3 \times 10^8$ | $1.8 \times 10^{10}$ |
| X1632 | 0.003 | $0.4 \times 10^8$ | $0.5 \times 10^{10}$ | 0.003 | $0.4 \times 10^8$ | $0.5 \times 10^{10}$ |
| CH119 | 0.005 | $0.6 \times 10^8$ | $0.9 \times 10^{10}$ | 0.004 | $0.5 \times 10^8$ | $0.7 \times 10^{10}$ |
| CE1176 | 0.008 | $1.0 \times 10^8$ | $1.4 \times 10^{10}$ | 0.007 | $0.9 \times 10^8$ | $1.2 \times 10^{10}$ |
| 25710 | 0.018 | $2.3 \times 10^8$ | $3.2 \times 10^{10}$ | 0.021 | $2.6 \times 10^8$ | $3.7 \times 10^{10}$ |
| BJOX2000 | 0.014 | $1.8 \times 10^8$ | $2.5 \times 10^{10}$ | 0.012 | $1.5 \times 10^8$ | $2.1 \times 10^{10}$ |
| CEO217 | 0.006 | $0.8 \times 10^8$ | $1.1 \times 10^{10}$ | 0.005 | $0.6 \times 10^8$ | $0.9 \times 10^{10}$ |
| CNE8 | 0.020 | $2.5 \times 10^8$ | $3.5 \times 10^{10}$ | 0.028 | $3.5 \times 10^8$ | $4.9 \times 10^{10}$ |
| X2278 | 0.017 | $2.1 \times 10^8$ | $3.0 \times 10^{10}$ | 0.018 | $2.3 \times 10^8$ | $3.2 \times 10^{10}$ |
| 246F3 | 0.010 | $1.3 \times 10^8$ | $1.8 \times 10^{10}$ | 0.011 | $1.4 \times 10^8$ | $1.9 \times 10^{10}$ |
| 398F1 | 0.045 | $5.6 \times 10^8$ | $7.9 \times 10^{10}$ | 0.013 | $1.6 \times 10^8$ | $2.3 \times 10^{10}$ |
| **Geometric mean** | **0.011** | $\mathbf{1.3 \times 10^8}$ | $\mathbf{1.9 \times 10^{10}}$ | **0.010** | $\mathbf{1.2 \times 10^8}$ | $\mathbf{1.7 \times 10^{10}}$ |

**Table S1. $IC_{50}$s for CD4-VLPs and CD4-CCR5-VLPs against a panel of HIV-1 strains.** In vitro neutralization of HIV-$1_{YU2}$ and a global panel of 12 HIV-1 Env reference strains (13). $IC_{50}$s against each strain and the geometric mean $IC_{50}$s for CD4-VLPs and CD4-CCR5-VLPs are presented in µg of p24/mL. $IC_{50}$ VLP concentrations were derived from p24 concentrations using the following equation: 1 ng p24 = $1.25 \times 10^7$ VLPs (14, 15), which assumes that each VLP contains 2,000 copies of p24 (4, 5). Since each VLP displays an average of ~140 copies of CD4 (Fig. S2), $IC_{50}$ concentrations of VLP-associated CD4 molecules were calculated by multiplying the number of VLPs by 140.

| | IC$_{50}$ | | | | | |
|---|---|---|---|---|---|---|
| **HIV Strain** | **sCD4 (µg/mL)** | **sCD4 (CD4 copies/mL)** | **CD4-Ig (µg/mL)** | **CD4-Ig (CD4 copies/mL)** | **3BNC117 (µg/mL)** | **3BNC117 (Fab copies/mL)** |
| YU2 | 0.26 | $6.0 \times 10^{12}$ | 0.93 | $1.1 \times 10^{13}$ | 0.016 | $1.3 \times 10^{11}$ |
| CNE55 | 18.8 | $4.4 \times 10^{14}$ | >50 | $6.0 \times 10^{14}$ | 0.14 | $1.1 \times 10^{12}$ |
| Tro11 | >50 | $1.2 \times 10^{15}$ | >50 | $6.0 \times 10^{14}$ | 0.034 | $2.7 \times 10^{11}$ |
| X1632 | 8.6 | $2.0 \times 10^{14}$ | 10.5 | $1.3 \times 10^{14}$ | 10.8 | $8.7 \times 10^{13}$ |
| CH119 | 2.5 | $5.8 \times 10^{13}$ | 30.4 | $3.7 \times 10^{14}$ | 14.0 | $1.1 \times 10^{14}$ |
| CE1176 | 6.8 | $1.6 \times 10^{14}$ | 31.3 | $3.8 \times 10^{14}$ | 0.12 | $9.6 \times 10^{11}$ |
| 25710 | 3.3 | $7.6 \times 10^{13}$ | 9.9 | $1.2 \times 10^{14}$ | 0.22 | $1.8 \times 10^{12}$ |
| BJOX2000 | 0.96 | $2.2 \times 10^{13}$ | 8.0 | $9.6 \times 10^{13}$ | >50 | $4.0 \times 10^{14}$ |
| CEO217 | 11.1 | $2.6 \times 10^{14}$ | 37.0 | $4.5 \times 10^{14}$ | 0.04 | $3.2 \times 10^{11}$ |
| CNE8 | >50 | $1.2 \times 10^{15}$ | >50 | $6.0 \times 10^{14}$ | 0.15 | $1.2 \times 10^{12}$ |
| X2278 | 12.3 | $2.8 \times 10^{14}$ | 22.3 | $2.7 \times 10^{14}$ | 0.011 | $8.8 \times 10^{10}$ |
| 246F3 | 6.7 | $1.6 \times 10^{14}$ | >50 | $6.0 \times 10^{14}$ | 0.14 | $1.1 \times 10^{12}$ |
| 398F1 | >50 | $1.2 \times 10^{15}$ | >50 | $6.0 \times 10^{14}$ | 0.092 | $7.4 \times 10^{11}$ |
| **Geometric mean** | **10.0** | **$2.3 \times 10^{14}$** | **27.7** | **$3.3 \times 10^{14}$** | **0.31** | **$2.5 \times 10^{12}$** |

| **µg/mL** | **0.01 – 0.1** | **0.1 – 1** | **1 - 10** | **10 - 50** | **> 50** |
|---|---|---|---|---|---|

**Table S2. IC$_{50}$s for sCD4, CD4-Ig, and 3BNC117 against a panel of HIV-1 strains.** In vitro neutralization of HIV-1$_{YU2}$ and a global panel of 12 HIV-1 Env reference strains (13) by sCD4, CD4-Ig, and 3BNC117. IC$_{50}$s against each strain and geometric mean IC$_{50}$s are shown in µg/mL. Colors indicate IC$_{50}$ ranges. IC$_{50}$s are also presented in CD4 copies/mL for sCD4 D1D2 (26 kDa) and CD4-Ig (100 kDa), and Fab copies/mL for 3BNC117 (150 kDa). Concentrations of CD4/Fab copies were derived by converting IC$_{50}$s into molar concentrations and then calculating the respective numbers of inhibitor molecules. The numbers of inhibitor molecules were then multiplied by a factor of one (sCD4) or two (CD4-Ig, 3BNC117) depending on whether a single inhibitor molecule contains one or two CD4/Fab copies.

| | $IC_{50}$ | | | | |
|---|---|---|---|---|---|
| **Patient Isolate** | **CD4-VLPs (VLPs/mL)** | **3BNC117 (µg/mL)<br>CD4bs** | **PGDM1400 (µg/mL)<br>V1V2** | **10E8 (µg/mL)<br>MPER** | **8ANC195 (µg/mL)<br>gp120-gp41 interface** |
| 601 Wk23 MQ19 | $2.5 \times 10^8$ | >50 | 7.3 | 11.3 | >50 |
| 605 Wk23 MK10 | $3.3 \times 10^8$ | 2.9 | >50 | 2.2 | 5.5 |

| **µg/mL** | **0.01 – 0.1** | **0.1 – 1** | **1 - 10** | **10 - 50** | **> 50** |
|---|---|---|---|---|---|

**Table S3. $IC_{50}$s for CD4-VLPs and various bNAbs against HIV-1 isolates obtained from infected individuals.** $IC_{50}$s are shown in VLPs/mL for CD4-VLPs and in µg/mL for the HIV-1 bNAbs 3BNC117, PGDM1400, 10E8, and 8ANC195. Epitopes on HIV-1 Env are listed for each bNAb (16). CD4bs = CD4 binding site and MPER = membrane-proximal external region. The colors indicate different $IC_{50}$ ranges for bNAbs.

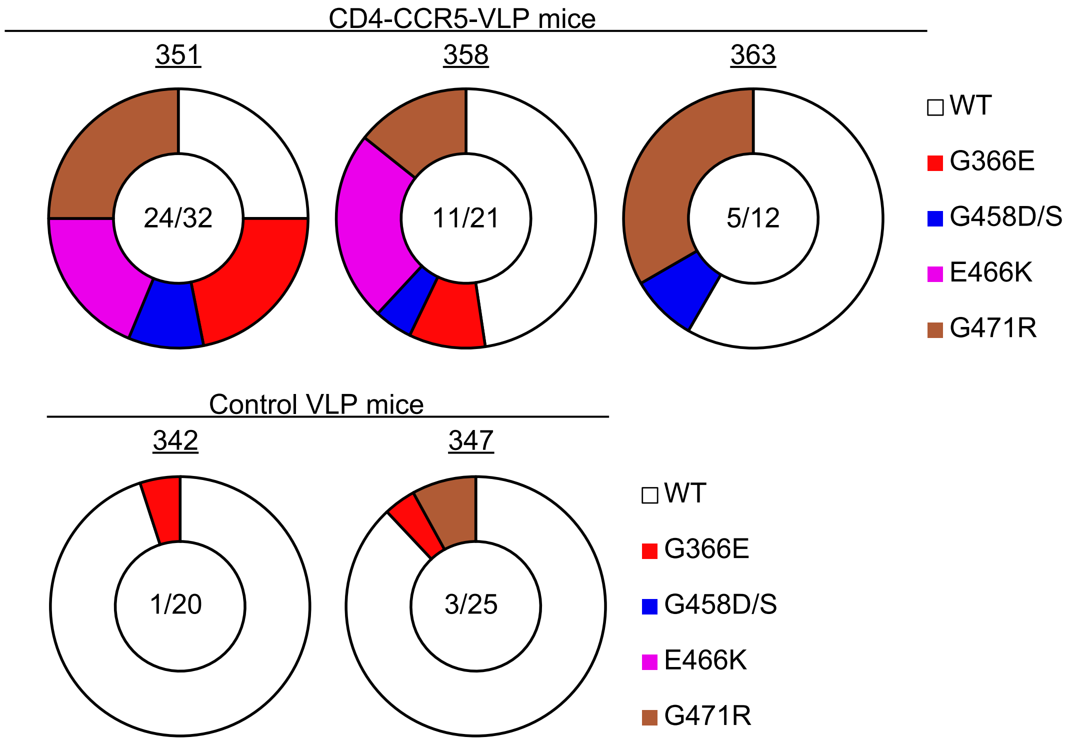


**Fig. S5. CD4-CCR5-VLP treatment selects mutations in HIV-1$_{YU2}$-infected humanized mice.** Pie charts showing the frequency of recurrent HIV-1$_{YU2}$ *env* mutations obtained from individual CD4-CCR5-VLP-treated (#351, #358, #363) and control-VLP-treated hu-mice (#342, #347). The slices are proportional to the number of sequences that contained the indicated substitutions. White slices represent the number of sequences that lacked any recurrent mutations. The numbers in the center denote the total number of sequences that contained recurrent mutations over the total number of sequences obtained for each animal.

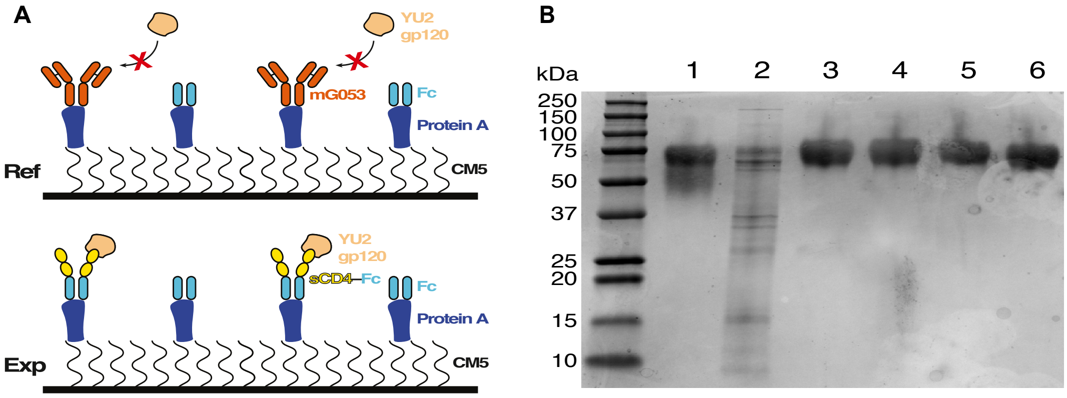


**Fig. S6. SPR binding analysis of YU2 gp120 mutants.** (**A**) Schematic showing the experimental setup for SPR binding assays. A non-HIV-1-binding IgG mG053 (reference) or CD4-Ig (experiment) were immobilized on protein A-coated CM5 biosensor chips followed by injection of a concentration series of monomeric YU2 gp120. (**B**) SDS-PAGE analysis of expressed $YU2_{wt}$ (lane 1), $YU2_{G366E}$ (lane 2), $YU2_{D457E}$ (lane 3), $YU2_{G458D}$ (lane 4), $YU2_{E466K}$ (lane 5), and $YU2_{G471R}$ (lane 6) gp120 proteins.

| Analyte | Capture protein | $k_a$ ($10^5$ $M^{-1}s^{-1}$) | SE($k_a$)($10^1$ $M^{-1}s^{-1}$) | $k_d$ ($10^{-4}s^{-1}$) | SE($k_d$) ($10^{-7}s^{-1}$) | $K_D$ (nM) |
|---|---|---|---|---|---|---|
| $YU2_{WT}$ gp120 | CD4-Ig | 1.1 | 8.6 | 1.1 | 14.0 | 1.0 |
| $YU2_{G458D}$ gp120 | CD4-Ig | 2.0 | 1.2 | 12.1 | 17.0 | 6.0 |
| $YU2_{E466K}$ gp120 | CD4-Ig | 1.6 | 5.2 | 2.7 | 7.9 | 1.7 |
| $YU2_{G471R}$ gp120 | CD4-Ig | 1.4 | 4.6 | 2.1 | 7.2 | 1.6 |
| $YU2_{G366E}$ gp120 | ND | ND | ND | ND | ND | ND |

**Table S4. $K_D$, $k_a$, $k_d$ values for binding of YU2 gp120 variants to CD4-Ig.** Results from SPR binding assays of CD4-Ig with $YU2_{wt}$ and YU2 variant gp120 proteins (see Fig. 5A). The standard errors (SEs) of the fits were as follows: $YU2_{WT}$ gp120 → CD4-Ig ($K_D$: 1.0 nM; SE: 12.8 pM), $YU2_{G458D}$ gp120 → CD4-Ig ($K_D$: 6.0 nM; SE: 9.1 pM), $YU2_{E466K}$ gp120 → CD4-Ig ($K_D$: 1.7 nM; SE: 5.0 pM), and $YU2_{G471R}$ gp120 → CD4-Ig ($K_D$: 1.6 nM; SE: 5.4 pM).

| | $IC_{50}$ | | | | | | | |
|---|---|---|---|---|---|---|---|---|
| **HIV Strain** | **CD4-VLPs (VLPs/mL)** | **Fold Change** | **CD4-CCR5-VLPs (VLPs/mL)** | **Fold Change** | **sCD4 (µg/mL)** | **Fold Change** | **CD4-Ig (µg/mL)** | **Fold Change** |
| YU2 | $2.5 \times 10^8$ | | $2.3 \times 10^8$ | | 0.29 | | 1.07 | |
| $YU2_{G366E}$ | $1.9 \times 10^8$ | 0.8 | $2.0 \times 10^8$ | 0.9 | 8.3 | 28.6 | 28.5 | 26.6 |
| $YU2_{G458D}$ | $3.0 \times 10^8$ | 1.2 | $3.5 \times 10^8$ | 1.6 | 4.3 | 14.8 | 40.0 | 37.4 |
| $YU2_{E466K}$ | $1.8 \times 10^8$ | 0.7 | $2.8 \times 10^8$ | 1.2 | 7.9 | 27.2 | 32.2 | 30.1 |
| $YU2_{G471R}$ | $2.6 \times 10^8$ | 1.1 | $2.5 \times 10^8$ | 1.1 | 9.0 | 31.0 | 34.2 | 32.0 |

| **µg/mL** | **0.01 – 0.1** | **0.1 – 1** | **1 - 10** | **10 - 50** |
|---|---|---|---|---|

**Table S5. $IC_{50}$s for CD4-VLPs, CD4-CCR5-VLPs, sCD4, and CD4-Ig against $YU2_{wt}$ and YU2 mutant pseudoviruses.** $IC_{50}$s are shown in VLPs/mL for CD4-VLPs and CD4-CCR5-VLPs and in µg/mL for sCD4 and CD4-Ig. The fold change was calculated by dividing a YU2 variant $IC_{50}$ by the $YU2_{wt}$ $IC_{50}$. The colors indicate $IC_{50}$ ranges for sCD4 and CD4-Ig.

## Bibliography


1. R. W. Sanders *et al.*, A next-generation cleaved, soluble HIV-1 Env trimer, BG505 SOSIP.664 gp140, expresses multiple epitopes for broadly neutralizing but not non-neutralizing antibodies. *PLoS Pathog* **9**, e1003618 (2013).
2. A. P. West, Jr. *et al.*, Evaluation of CD4-CD4i antibody architectures yields potent, broadly cross-reactive anti-human immunodeficiency virus reagents. *J Virol* **84**, 261-269 (2010).
3. H. Wang *et al.*, Cryo-EM structure of a CD4-bound open HIV-1 envelope trimer reveals structural rearrangements of the gp120 V1V2 loop. *Proc Natl Acad Sci U S A* **113**, E7151-E7158 (2016).
4. L. A. Carlson *et al.*, Three-dimensional analysis of budding sites and released virus suggests a revised model for HIV-1 morphogenesis. *Cell Host Microbe* **4**, 592-599 (2008).
5. N. L. Goicochea *et al.*, Structure and stoichiometry of template-directed recombinant HIV-1 Gag particles. *J Mol Biol* **410**, 667-680 (2011).
6. D. N. Mastronarde, Automated electron microscope tomography using robust prediction of specimen movements. *J Struct Biol* **152**, 36-51 (2005).
7. W. J. H. Hagen, W. Wan, J. A. G. Briggs, Implementation of a cryo-electron tomography tilt-scheme optimized for high resolution subtomogram averaging. *J Struct Biol* **197**, 191-198 (2017).
8. J. R. Kremer, D. N. Mastronarde, J. R. McIntosh, Computer visualization of three-dimensional image data using IMOD. *J Struct Biol* **116**, 71-76 (1996).
9. L. Scharf *et al.*, Broadly Neutralizing Antibody 8ANC195 Recognizes Closed and Open States of HIV-1 Env. *Cell* **162**, 1379-1390 (2015).
10. J. A. Horwitz *et al.*, Non-neutralizing Antibodies Alter the Course of HIV-1 Infection In Vivo. *Cell* **170**, 637-648 e610 (2017).
11. J. F. Salazar-Gonzalez *et al.*, Deciphering human immunodeficiency virus type 1 transmission and early envelope diversification by single-genome amplification and sequencing. *J Virol* **82**, 3952-3970 (2008).
12. J. C. Lorenzi *et al.*, Paired quantitative and qualitative assessment of the replication-competent HIV-1 reservoir and comparison with integrated proviral DNA. *Proc Natl Acad Sci U S A* **113**, E7908-E7916 (2016).
13. A. deCamp *et al.*, Global panel of HIV-1 Env reference strains for standardized assessments of vaccine-elicited neutralizing antibodies. *J Virol* **88**, 2489-2507 (2014).
14. J. S. Gach *et al.*, Human immunodeficiency virus type-1 (HIV-1) evades antibody-dependent phagocytosis. *PLoS Pathog* **13**, e1006793 (2017).
15. C. A. Vink *et al.*, Eliminating HIV-1 Packaging Sequences from Lentiviral Vector Proviruses Enhances Safety and Expedites Gene Transfer for Gene Therapy. *Mol Ther* **25**, 1790-1804 (2017).
16. L. E. McCoy, D. R. Burton, Identification and specificity of broadly neutralizing antibodies against HIV. *Immunol Rev* **275**, 11-20 (2017).
